# Supplementary material for: Analysis of medieval burials from Ibiza reveals genetic and pathogenic diversity during the Islamic period
Source: Nat Commun. 2026 Mar 26;17:2703. doi: 10.1038/s41467-026-70615-9 (PMC13021928; doi:10.1038/s41467-026-70615-9)
Supplement: Supplementary file 1 — Supplementary Information [file 41467_2026_70615_MOESM1_ESM.pdf]

# Supplemental Material For:

## Analysis of medieval burials from Ibiza reveals genetic and pathogenic diversity during the Islamic period

Ricardo Rodríguez-Varela[1,2*]; Zoé Pochon[1,2]; Alex Mas-Sandoval[3]; Reyhan Yaka[1,2]; Cesar A. Fortes-Lima[4,5]; Almudena García Rubio[6]; Nicholas Márquez-Grant[7,8]; Juanjo Marí[9]; Glenda Graziani[10]; Antoni Ferrer Abárzuza[11]; Mário Vicente[1,2]; Lander Lorca-Francisco[1]; Anna Linderholm[1,12]; Vendela k. Lagerholm[1,2]; Lara R. Arauna[13]; Patxi Pérez-Ramallo[14,15,16]; Maja Krzewińska[1,2]; Carina M. Schlebusch[17,18,5] Anders Götherström[1,2,*]

**Affiliations**

[1] Centre for Palaeogenetics, Stockholm, Sweden

[2] Department of Archaeology and Classical Studies, Stockholm University, Sweden

[3] Departamento de Historia, Geografía y Comunicación, Universidad de Burgos, Spain

[4] McKusick-Nathans Institute and Department of Genetic Medicine, Johns Hopkins University School of Medicine, Baltimore, Maryland, USA

[5] Human Evolution, Department of Organismal Biology, Evolutionary Biology Centre, Uppsala University, Uppsala, Sweden

[6] Aranzadi Science Society, San Sebastián, Spain

[7] Cranfield Forensic Institute, Cranfield University, UK

[8] School of Anthropology and Museum Ethnography, University of Oxford, UK

[9] Independent researcher, Ibiza, Spain

[10] Departament de Ciències de l'Antiguitat i Edat Mitjana. Universitat Autònoma de Barcelona, Spain

[11] Consell Insular de Formentera, Balearic Islands, Spain

[12] Department of Geological Sciences, Stockholm University, Stockholm, 10691, Sweden

[13] Departament de Biologia Evolutiva, Ecologia i Ciències Ambientals (BEECA), Facultat de Biologia, Universitat de Barcelona (UB), Barcelona, Spain

[14] Social History of Capitalism research group (SHOC), Vrije Universiteit Brussel (VUB), Brussels, Belgium

[15] Archaeology, Environmental Changes & Geo-Chemistry research group (AMGC), Vrije Universiteit Brussel (VUB), Brussels, Belgium

[16] Department of Coevolution of Land Use and Urbanisation, Max Planck Insitute of Geoanthropology, Jena, Germany

[17] Palaeo-Research Institute, University of Johannesburg, Johannesburg, South Africa

[18] Center for the Human Past, Department of Organismal Biology, Uppsala, Sweden

*Corresponding authors (ricardo.rodriguez.varela@arklab.su.se; anders.gotherstrom@arklab.su.se)

## Supplementary Discussion

### Radiocarbon dates and Marine reservoir

To assess potential marine offsets, we estimated marine dietary protein for all twelve (n = 12) directly dated individuals using the Bayesian mixing package simmr (R 4.5.0) [1]. Mixtures were the dentine-collagen δ13C and δ15N values for each individual; source baselines followed our previous approach: terrestrial herbivores (n = 32) from Gandía late-medieval herbivores [2], Zorita rabbits [3], and three medieval Ibiza fauna [4]; δ13C = −18.3 ± 2.2‰, δ15N = 6.3 ± 2.3‰) and a well-dated Mediterranean fish assemblage from Provence (n = 127; δ13C = −9.5 ± 2.7‰, δ15N = 10.6 ± 2.1‰). Across individuals, the marine-fish fraction ranges 17.7–34.7 % (mean across individuals 21.8, SD 1.4; median 20.0). We report per-individual posterior summaries in Supplementary Data 2.

All radiocarbon determinations were then calibrated in OxCal v4.4.4 [5] under two models per individual: a purely terrestrial IntCal20 calibration [6] and, a mixed IntCal20–Marine20 calibration that incorporates each person's simmr pmarine (mean ± SD) together with a regional marine offset ΔR = -42 ± 30 14C yr from Banyuls [7]. Complete 68.3% and 95.4% Highest Posterior Densities (HPD) for both models are given in Supplementary Data 2 (see also Supplementary Fig. 1).

Under the mixed model, s.197 is robustly post-1115 CE (HPD68.3%= 1165–1280 CE), whereas s.117 straddles the 1115 threshold (HPD68.3 = 1051–1264 CE): compatible with, but not conclusive for, a post-1115 placement. Most other individuals likewise straddle 1115 at 68.3%, and s.313 remains earlier (HPD68.3% = 896–1046 CE). Because the dated material is permanent molar dentine, formed decades before death in mature adulthood, dates of death would be expected to fall later than the dentine formation windows, which further supports a post-1115 placement for s.197 and increases the plausibility for s.117, while we acknowledge remaining uncertainty at 95.4%. Limitations include non-fully local/coevel fauna, wide diet posteriors for some individuals, and the use of dentine (formation ≠ death); we handle these by reporting both terrestrial and mixed calibrations and by framing all results probabilistically (HPDs). These results are overall consistent with the independent archaeological span proposed for the cemetery (ca. 950–1150 CE) [4], with one clearly early burial (s.313) and one clearly late burial (s.197), while the remainder fall broadly within the 11th–12th centuries when marine-diet uncertainty is considered.

### Metagenomics

<u>Investigation of specific pathogens with scores lower than 8</u>

Individual s.197 yielded a *Mycobacterium leprae* hit with an authentication score of 7 in one of six sequencing runs of the same library. Upon reviewing the KrakenUniq outputs from each run (Supplementary Data 7), we suspect that although all libraries were double-indexed, this signal likely results from index hopping. The affected run was sequenced at higher depth and included the capture-enriched library of sample s.313, which harboured a confirmed *M. leprae* infection. Another individual from the same run, despite very low coverage, also showed *M. leprae* reads, further supporting the likelihood of low-level cross-contamination.

Only one library was prepared for s.197 and sequenced across six lanes. Notably, only the lane containing the s.313 capture-enriched sample showed detectable *M. leprae* reads in s.197, as well as in other unrelated, low-coverage samples sequenced in the same lane. The contamination appears to be limited in the case of s.197, involving approximately 1,000 to 2,000 *M. leprae* reads.

This case highlights the importance of exercising caution when pooling capture-enriched and shotgun libraries for sequencing, particularly in studies focused on low-abundance pathogen detection.

*Mycobacterium leprae* reference genome dataset

The dataset of all published ancient *Mycobacterium leprae* genomes was extracted based on the AncientMetagenomeDir database using AMDirT [8,9] Ancient genomes of 42 ancient samples with a depth of ≥ 3 were included in the dataset [10–17] . Similarly, modern genomes of 139 samples with a depth of ≥ 3 were included in the dataset [18–24] .

## Supplementary Figures

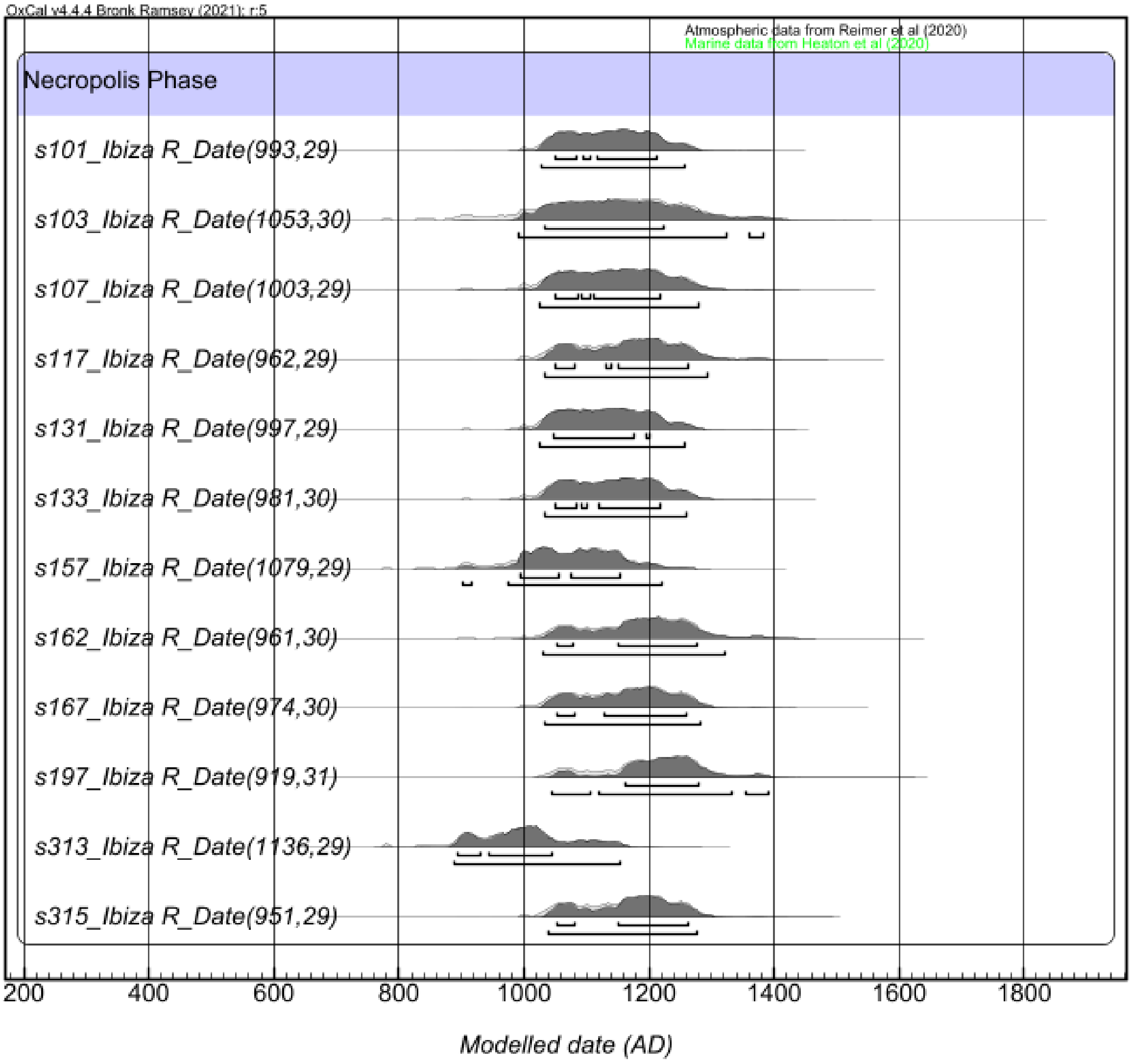


**Supplementary Fig. 1. Calibrated 14C probability distributions for all dated individuals.** Black bars mark 95.4 % credible intervals; shaded areas indicate posterior density.

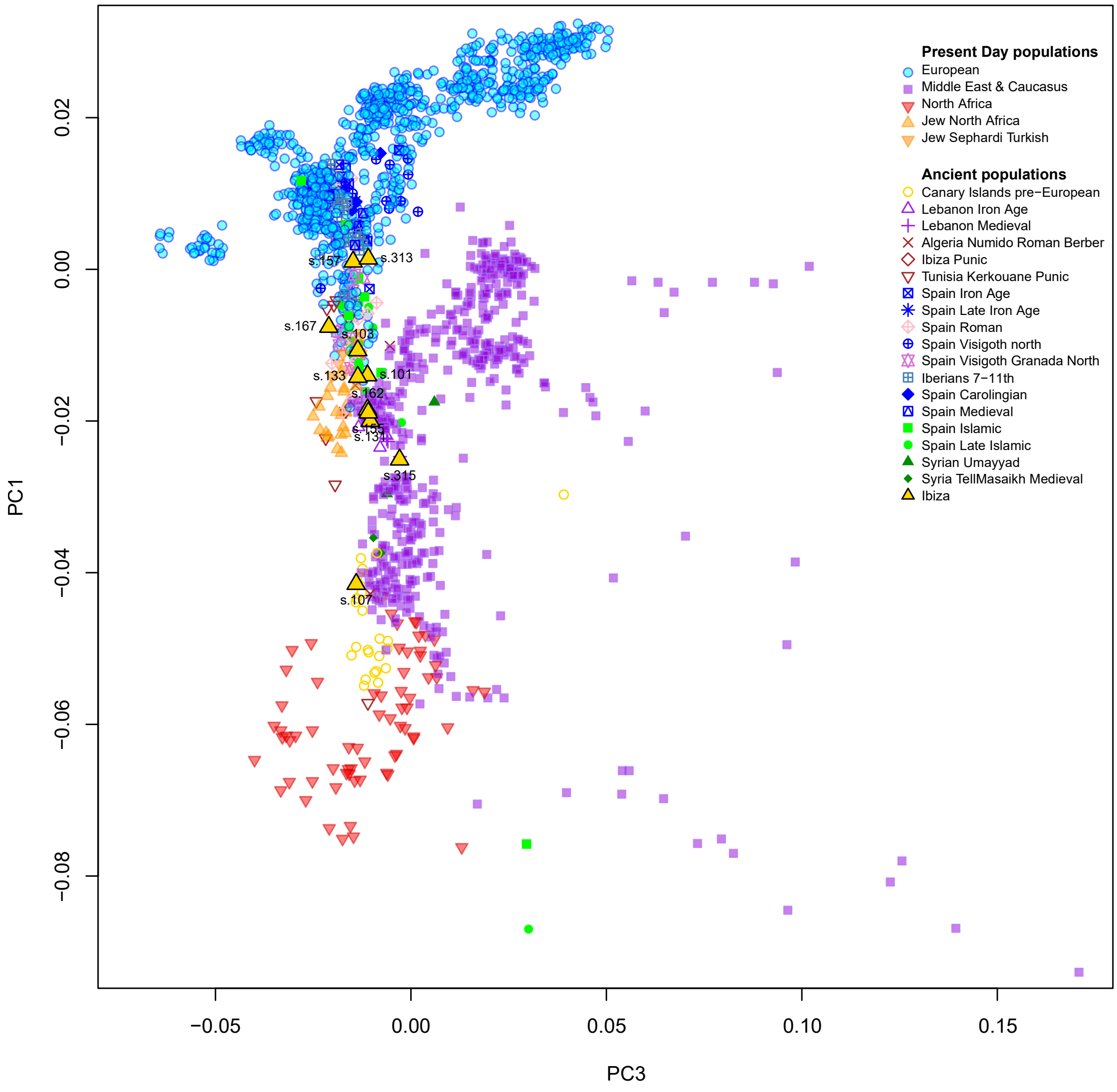


**Supplementary Fig. 2. Principal Component Analysis (PCA) of Eurasian populations with ancient DNA individuals projected.** Ancient individuals (excluding the two Sub-Saharan African samples) are projected onto PC1 and PC3, based on modern Eurasian genetic variation from the Human Origins (HO) dataset. Ancient genotypes are pseudo-haploid.

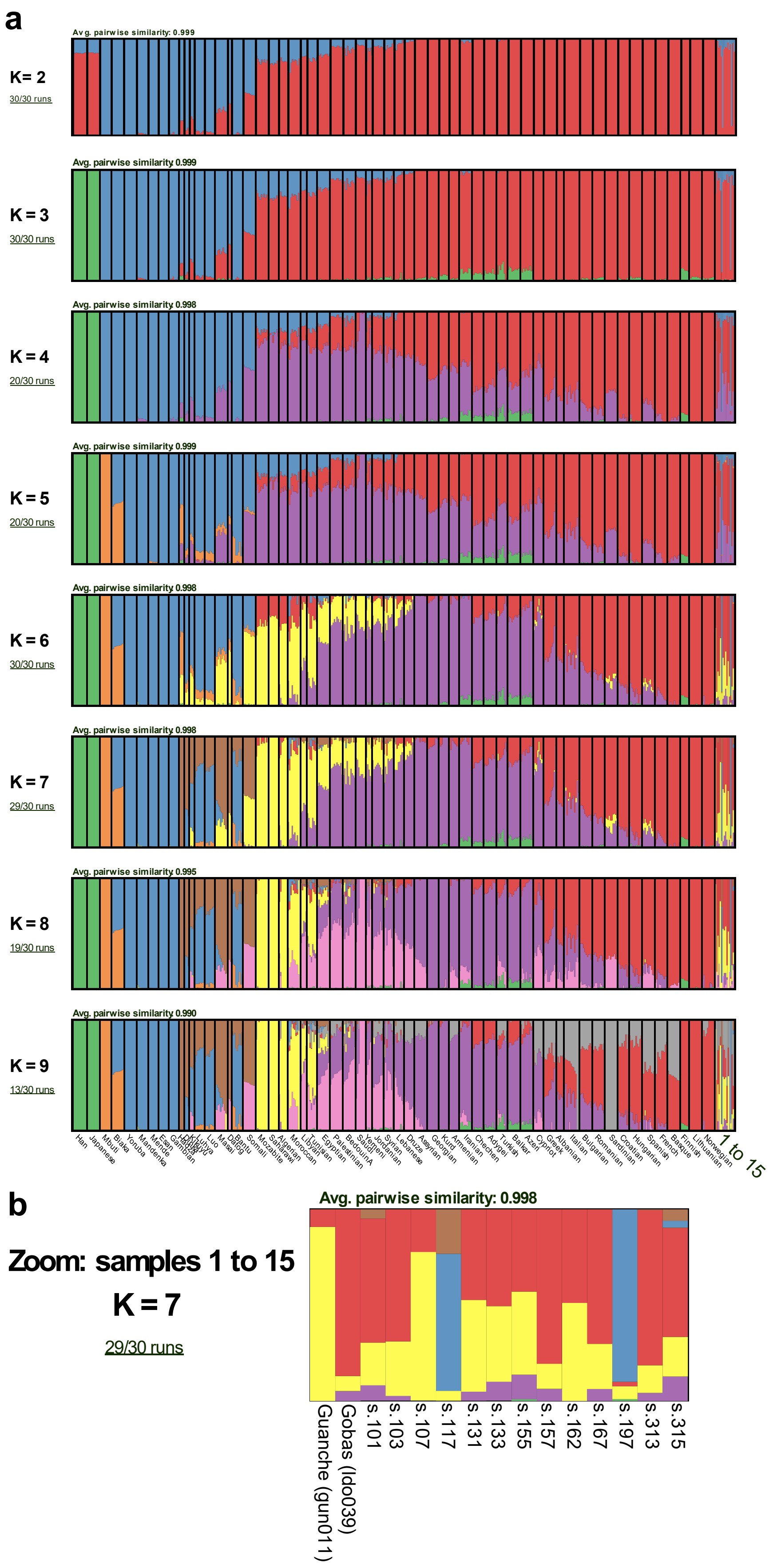


**Supplementary Fig. 3. Unsupervised ADMIXTURE Human Origins pseudohaploid dataset. a.** K=2 to k=9. 1 to 15: Guanche (gun011); Gobas (ldo039); s.101; s.103; s.107; s.117; s.131; s.133; s.155; s.157; s.162; s.167; s.197; s.313; s.315. **b**. Inset: zoom-in on the ancient individuals at K = 7.

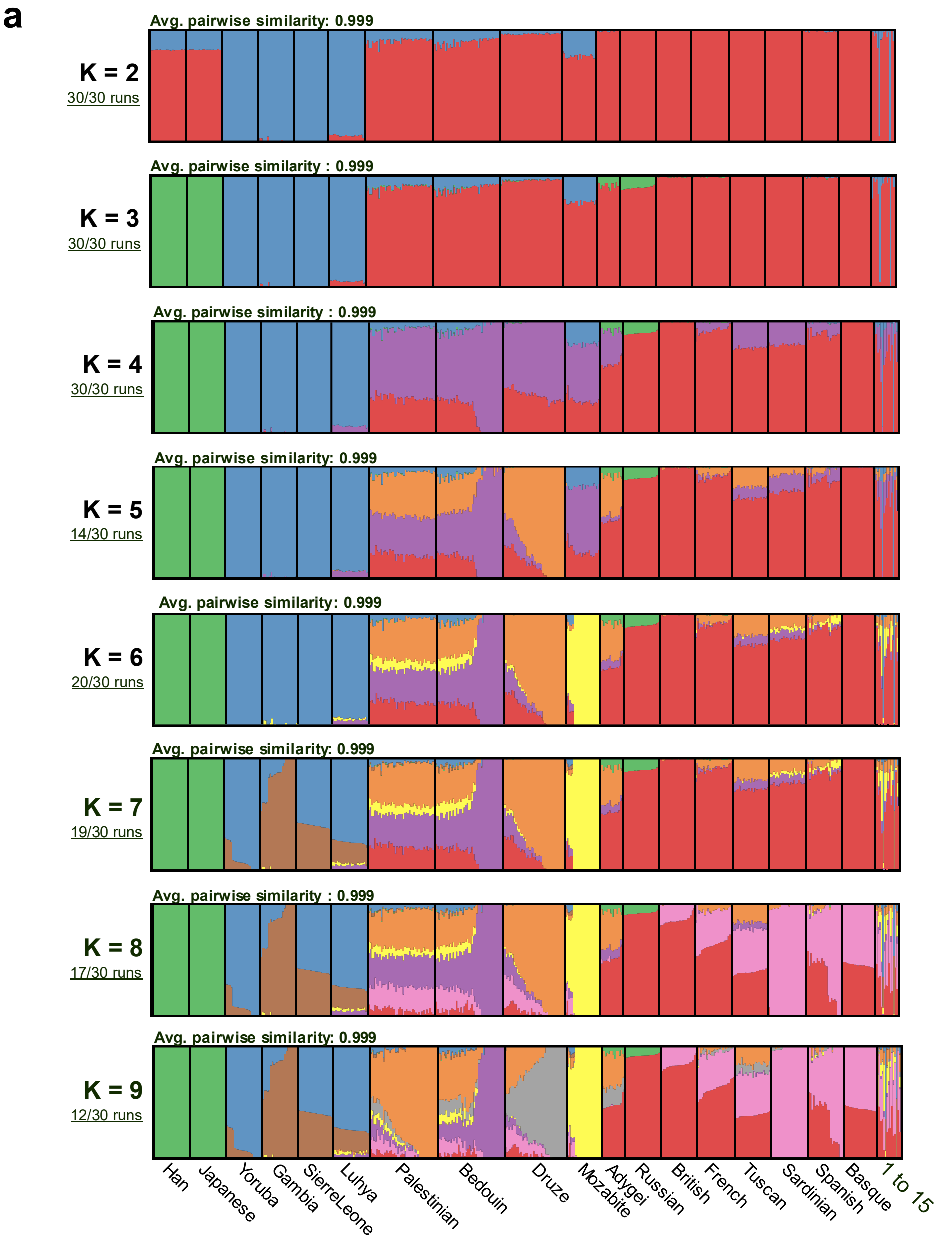


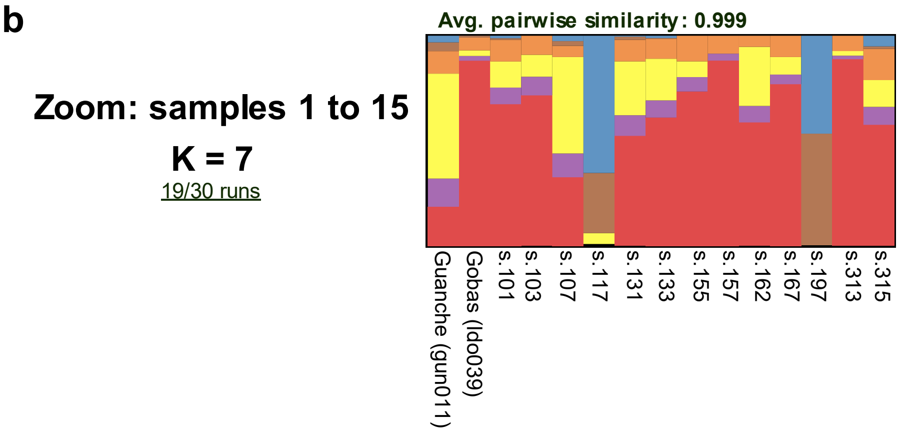


**Supplementary Fig. 4. Unsupervised ADMIXTURE. 1000 Genomes Project plus HDGP and imputed diploid dataset. a**. K=2 to K=9. 1 to 15: Guanche (gun011); Gobas (ldo039); s.101; s.103; s.107; s.117; s.131; s.133; s.155; s.157; s.162; s.167; s.197; s.313; s.315. **b.** Inset: zoom-in on the ancient individuals at K = 7.

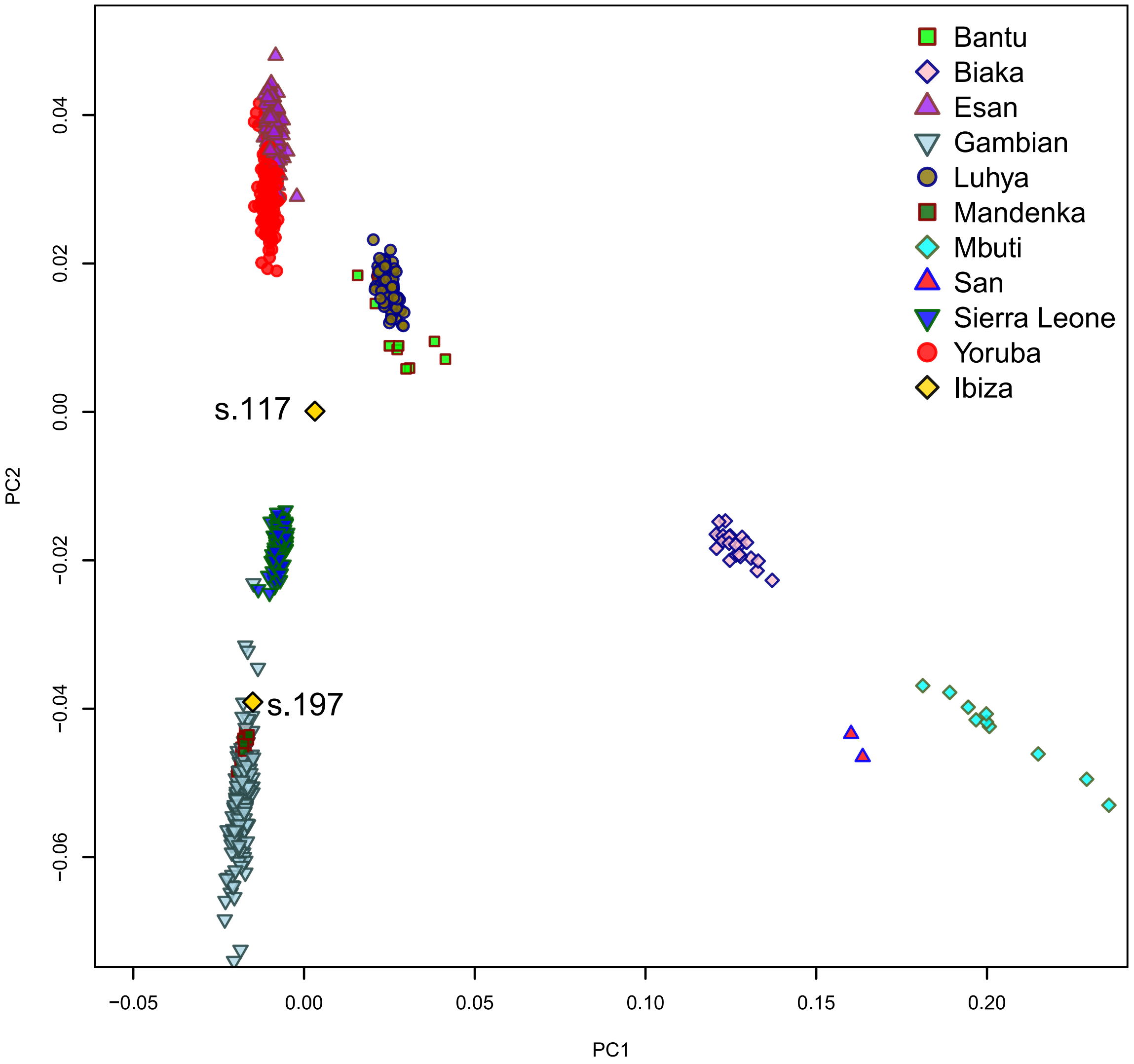


**Supplementary Fig. 5. First two principal components of the two ancient Sub-Saharan individuals projected onto modern sub-Saharan Africans included in the 1K-HGDP dataset.**

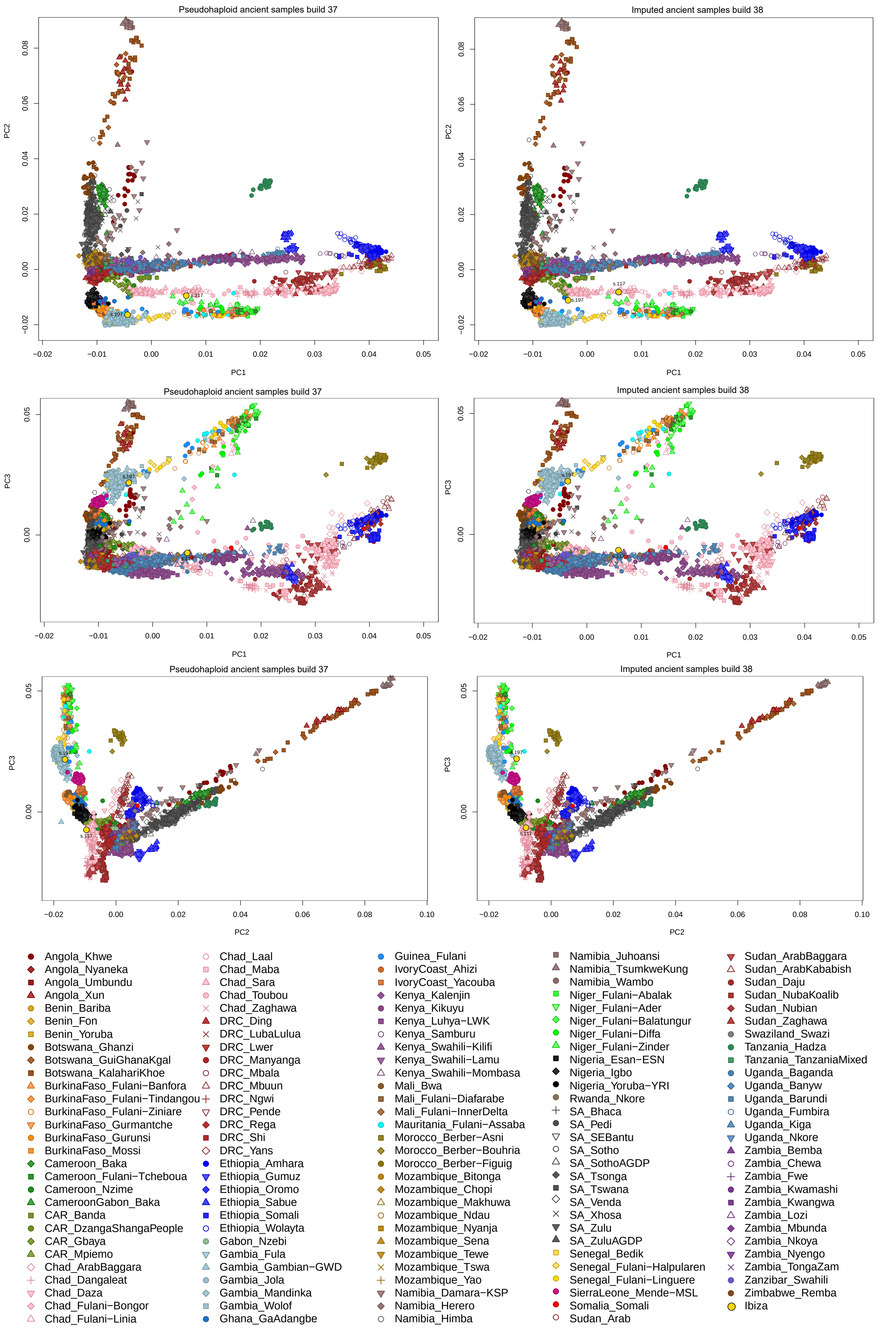


**Supplementary Fig. 6. Principal Component Analysis (PCA) showing the projection of ancient pseudo-haploid (left) and imputed diploid (right) Ibiza Sub-Saharan individuals onto modern African genetic variation with reference Africa1 dataset.**

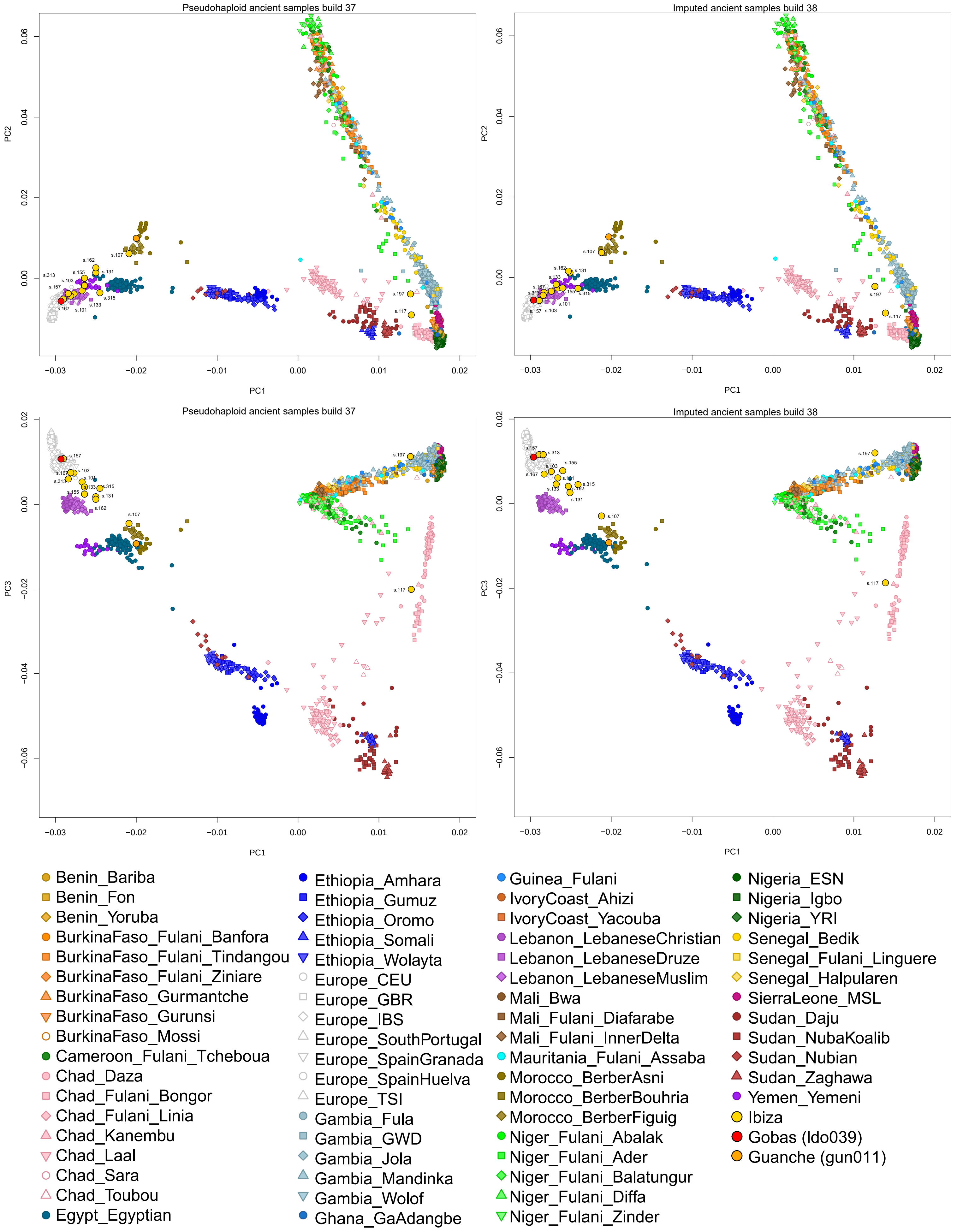


**Supplementary Fig. 7. Principal Component Analysis (PCA) showing the projection of ancient pseudo-haploid (left) and imputed diploid (right) Ibiza Sub-Saharan individuals onto modern African genetic variation with reference Africa2 dataset.**

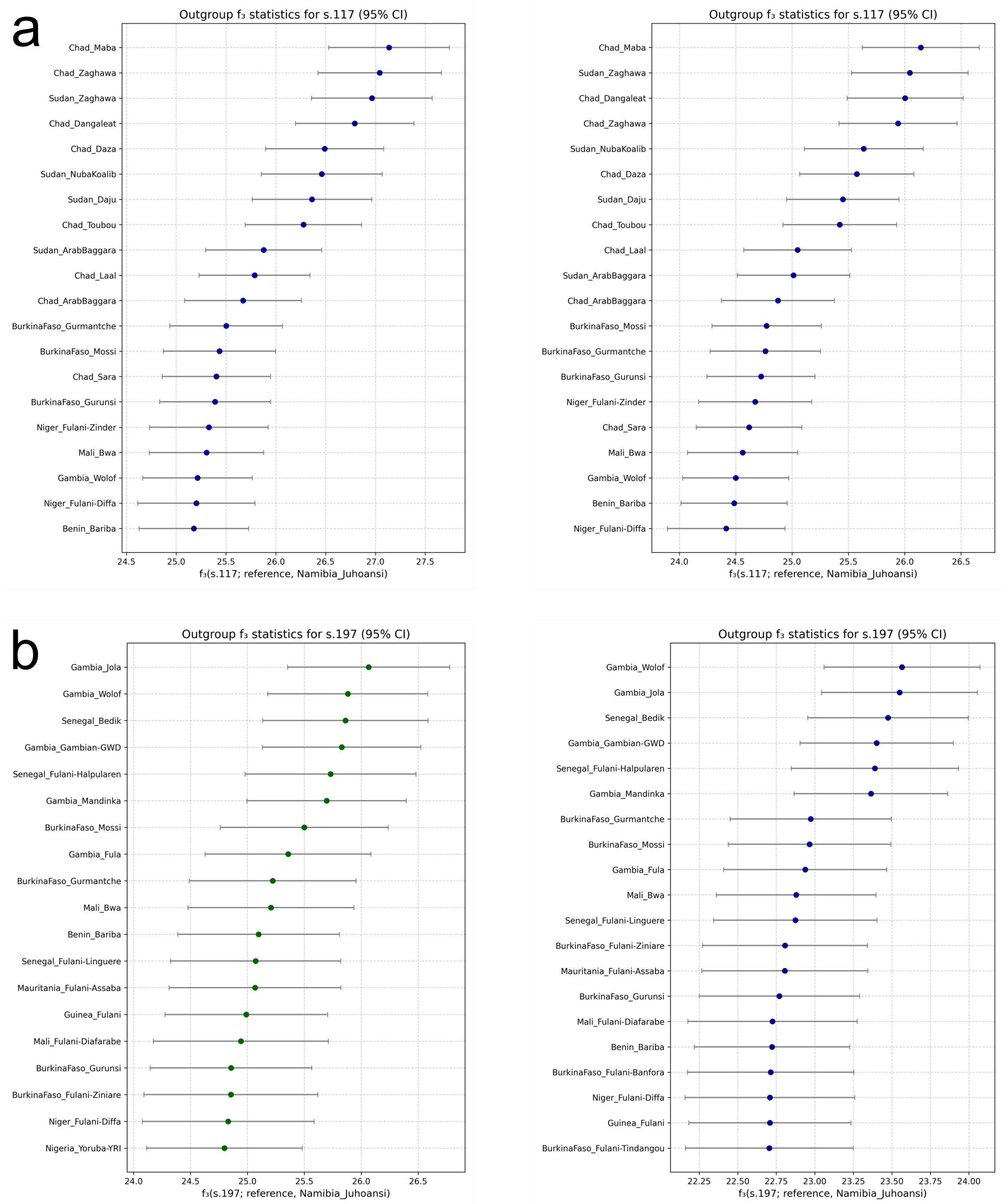


**Supplementary Fig. 8. Outgroup f3-statistics measuring shared genetic drift between sub-Saharan African ancient Ibiza individuals and modern African populations.** Each dot represents an f3-statistic of the form f3(Ibiza; Modern Population, Outgroup), with higher values indicating greater shared genetic ancestry. Error bars represent 95% confidence intervals. The outgroup used is the Namibian Ju'hoansi. Results are shown for two datasets: one using pseudohaploid ancient genotypes and the other using imputed diploid ancient genotypes. a. Individual s.117. b. Individual s.197.

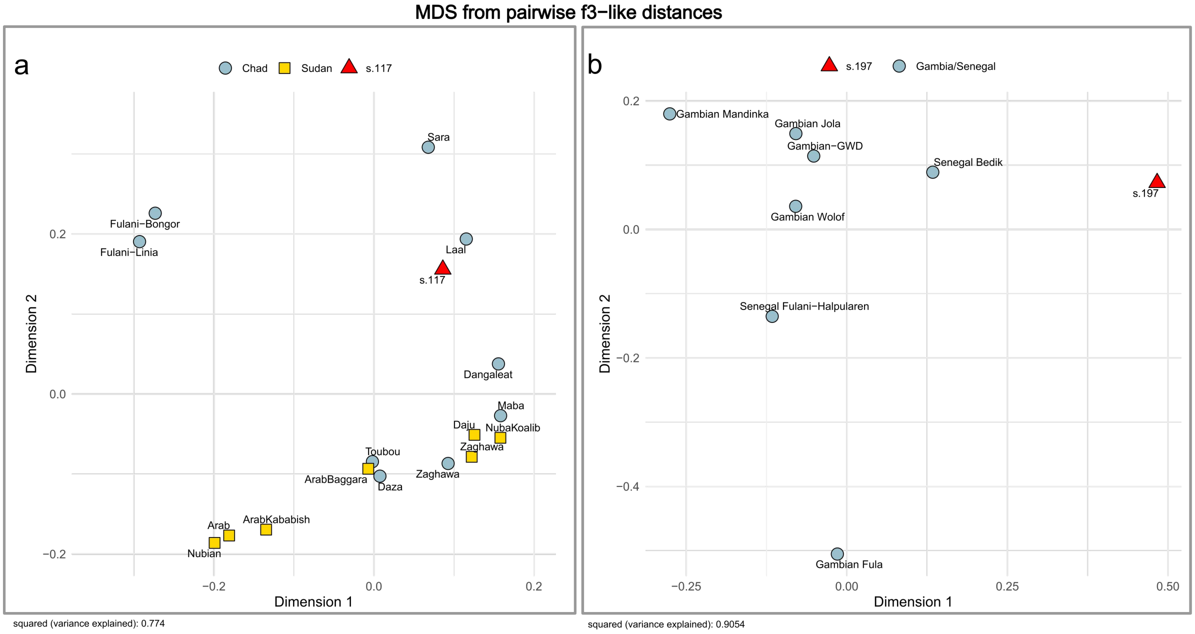


**Supplementary Fig. 9. Multidimensional scaling (MDS) plots based on pairwise outgroup $f_3$-statistics.** The outgroup used is the Namibian Ju'hoansi. **a**. MDS of individual s.117 and populations from Chad and Sudan, showing clustering of s.117 with groups from southern Chad (e.g., Laal). **b**. MDS of individual s.197 and populations from the Senegambia region, indicating affinities with several West African groups.

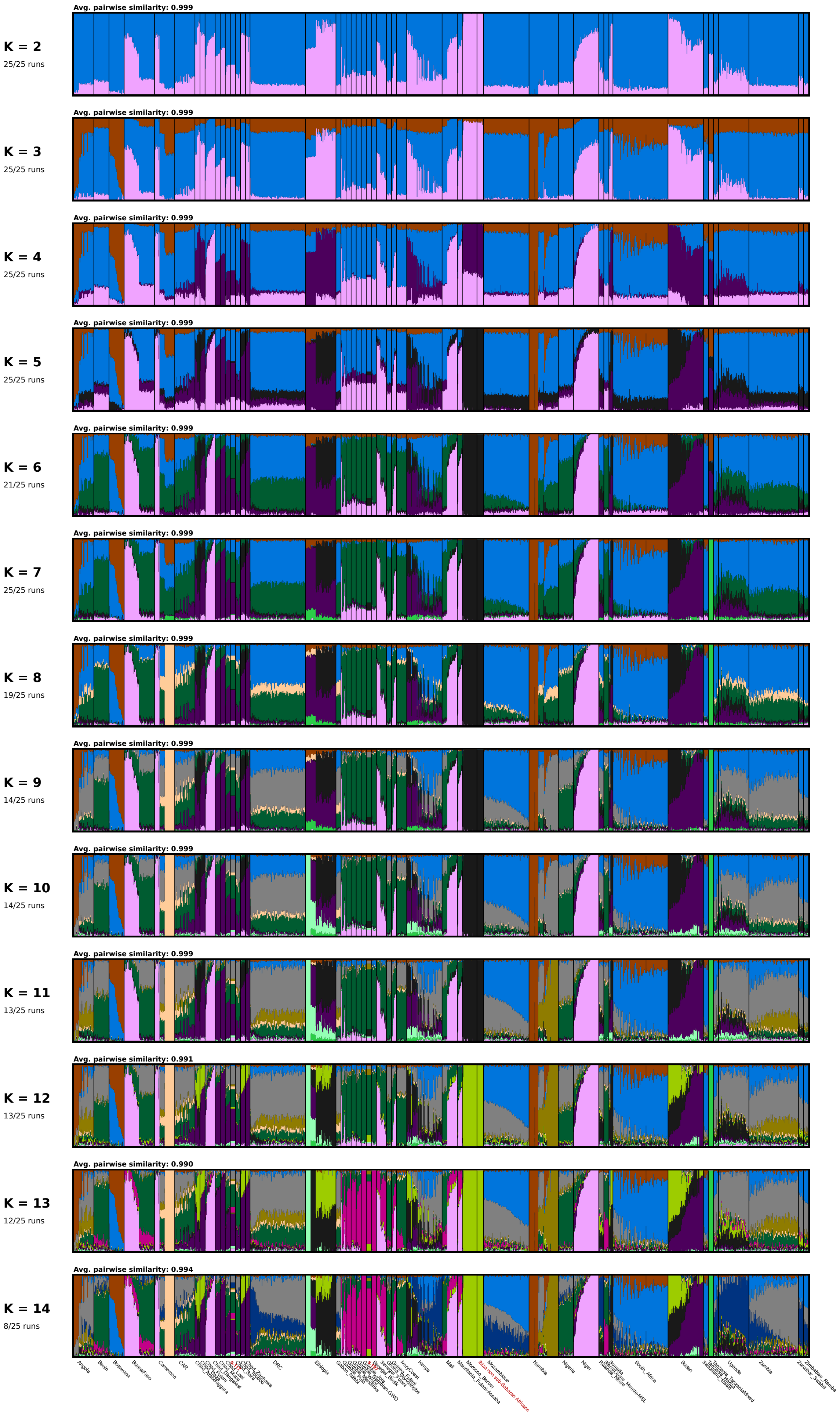


**Supplementary Fig. 10. Unsupervised ADMIXTURE analysis using Africa Dataset 1.** For visualization purposes, the two Sub-Saharan African ancient samples, s.117 and s.197, were replicated 10 times in the plot.

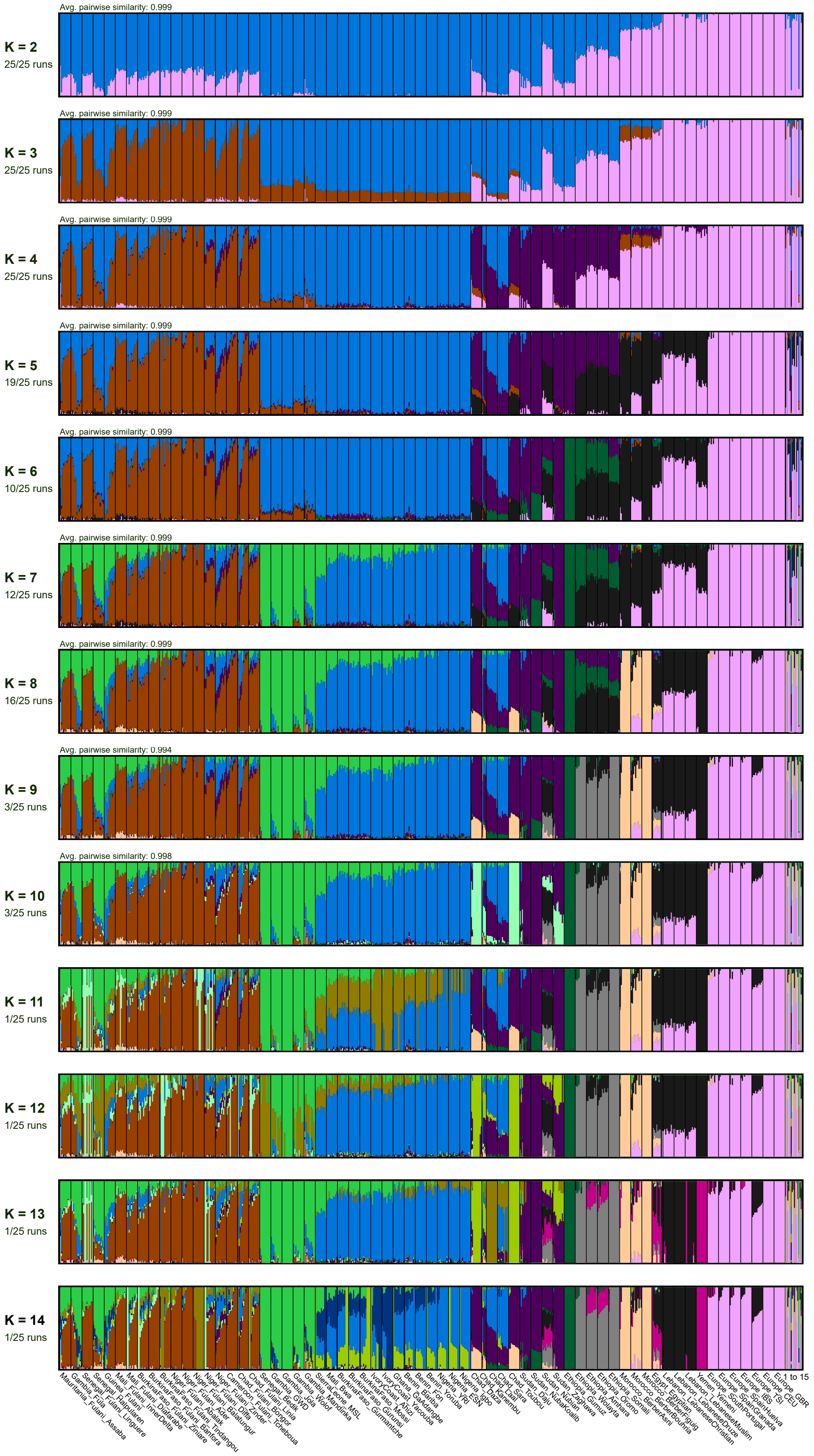


**Supplementary Fig. 11. Unsupervised ADMIXTURE analysis using Africa Dataset 2.** 1 to 15: Guanche (gun011); Gobas (ldo039); s.101; s.103; s.107; s.117; s.131; s.133; s.155; s.157; s.162; s.167; s.197; s.313; s.315.

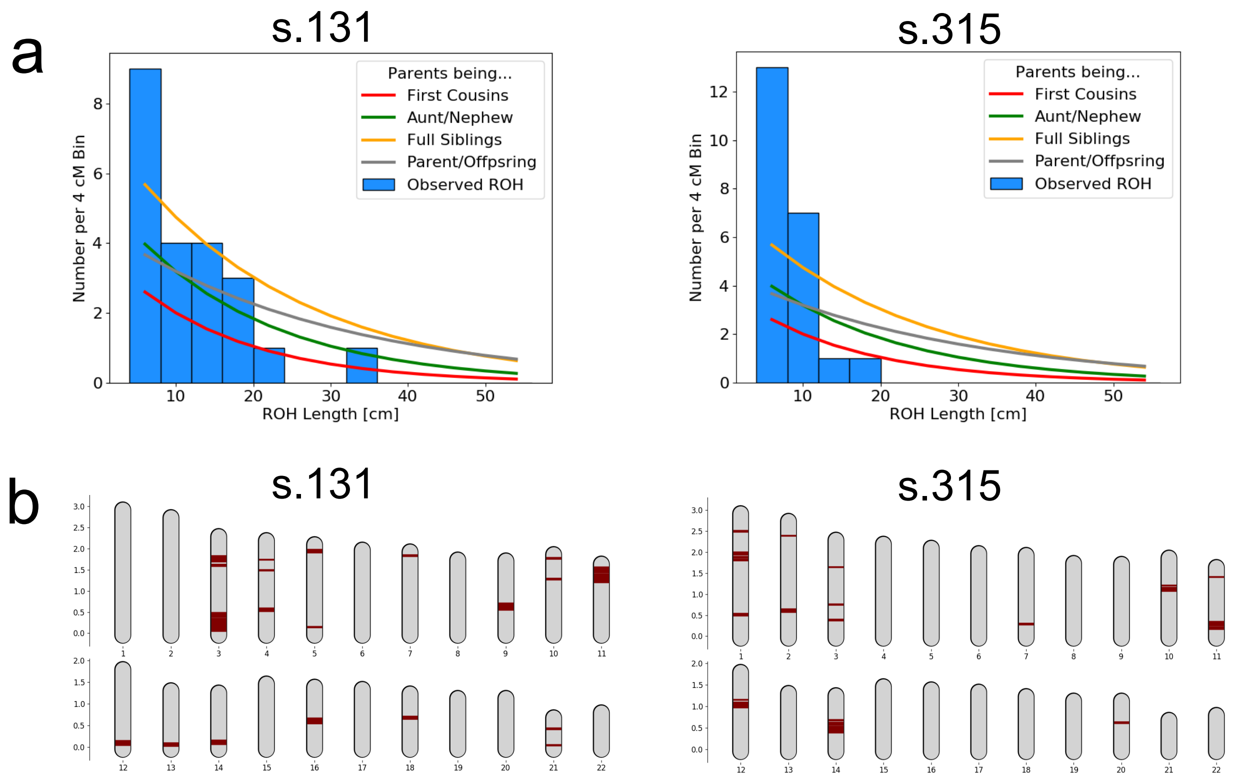


**Supplementary Fig. 12. Runs of homozygosity in the individual with the highest total ROH.** **a**. ROH lengths and expected density distributions for different degrees of parental relatedness. **b**. Chromosomal positions of the ROH fragments in this individual.

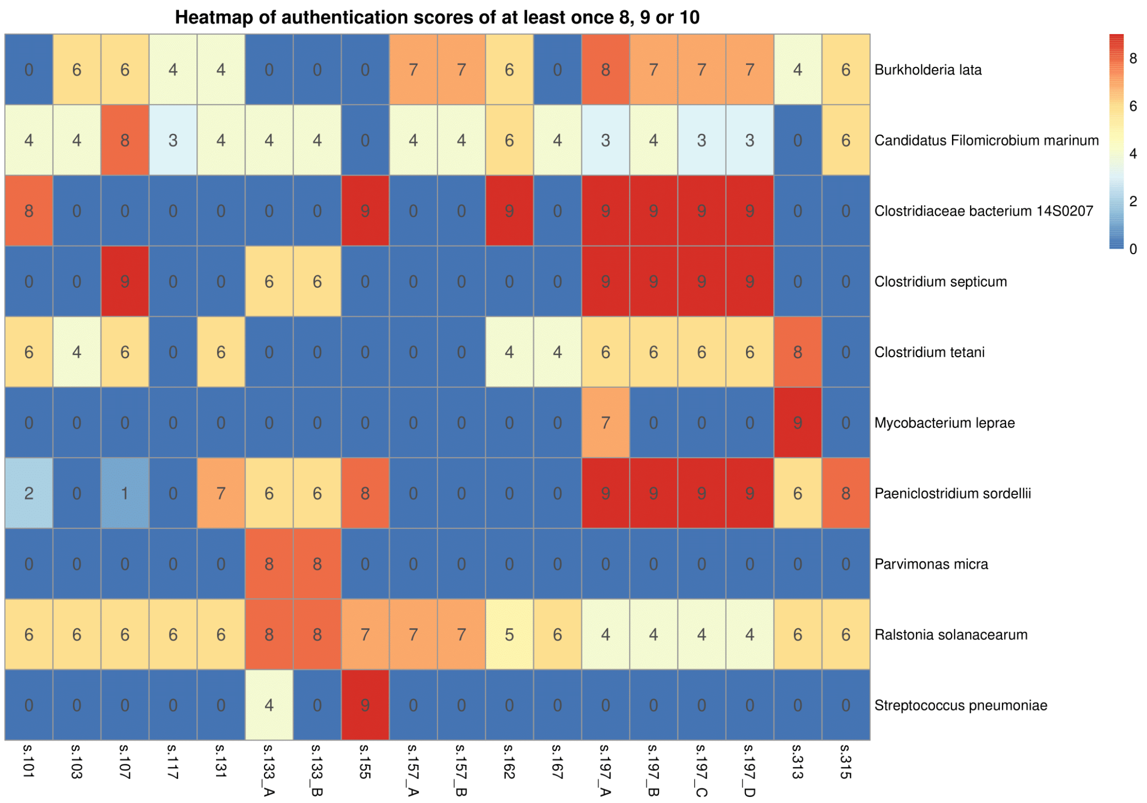


**Supplementary Fig. 13. Heatmap showing authentication scores ≥ 8 per microbe and per individual.** Scores reflect nine evaluation criteria: edit distance, ancient-read edit distance, terminal deamination (both ends), average read length, PMD score, average nucleotide identity (ANI), number of reads, and coverage evenness (which contributes two points). Values range from 1 to 10.

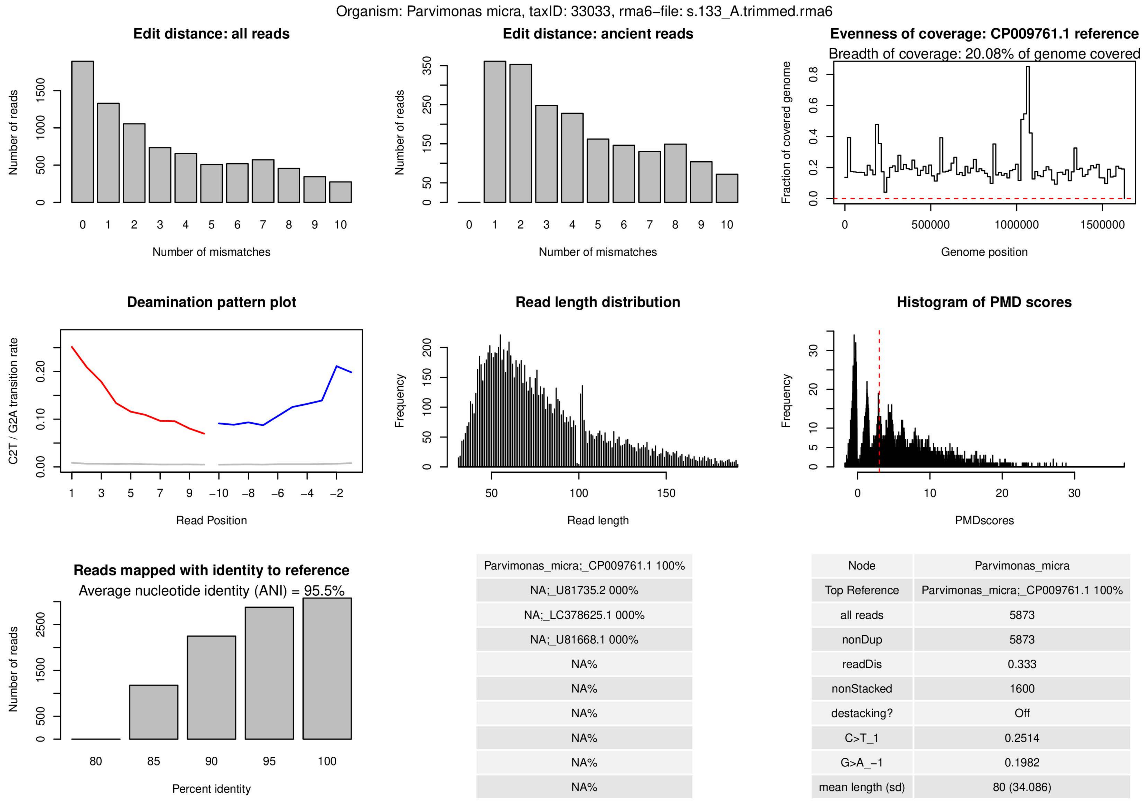


| |
|---|
| Parvimonas_micra;_CP009761.1 100% |
| NA;_U81735.2 000% |
| NA;_LC378625.1 000% |
| NA;_U81668.1 000% |
| NA% |
| NA% |
| NA% |
| NA% |
| NA% |
| NA% |

| | |
|---|---|
| Node | Parvimonas_micra |
| Top Reference | Parvimonas_micra;_CP009761.1 100% |
| all reads | 5873 |
| nonDup | 5873 |
| readDis | 0.333 |
| nonStacked | 1600 |
| destacking? | Off |
| C>T_1 | 0.2514 |
| G>A_-1 | 0.1982 |
| mean length (sd) | 80 (34.086) |

**Supplementary Fig. 14. Authentication plots for *Parvimonas micra* in individual s.133_A.** The panels display edit distance distributions (all vs ancient reads), genome coverage breadth and evenness, terminal deamination rates, read length distribution, PMD score histogram, and percent identity of mapped reads. A summary table includes top reference matches and mapping statistics. The overall authentication score is 8.

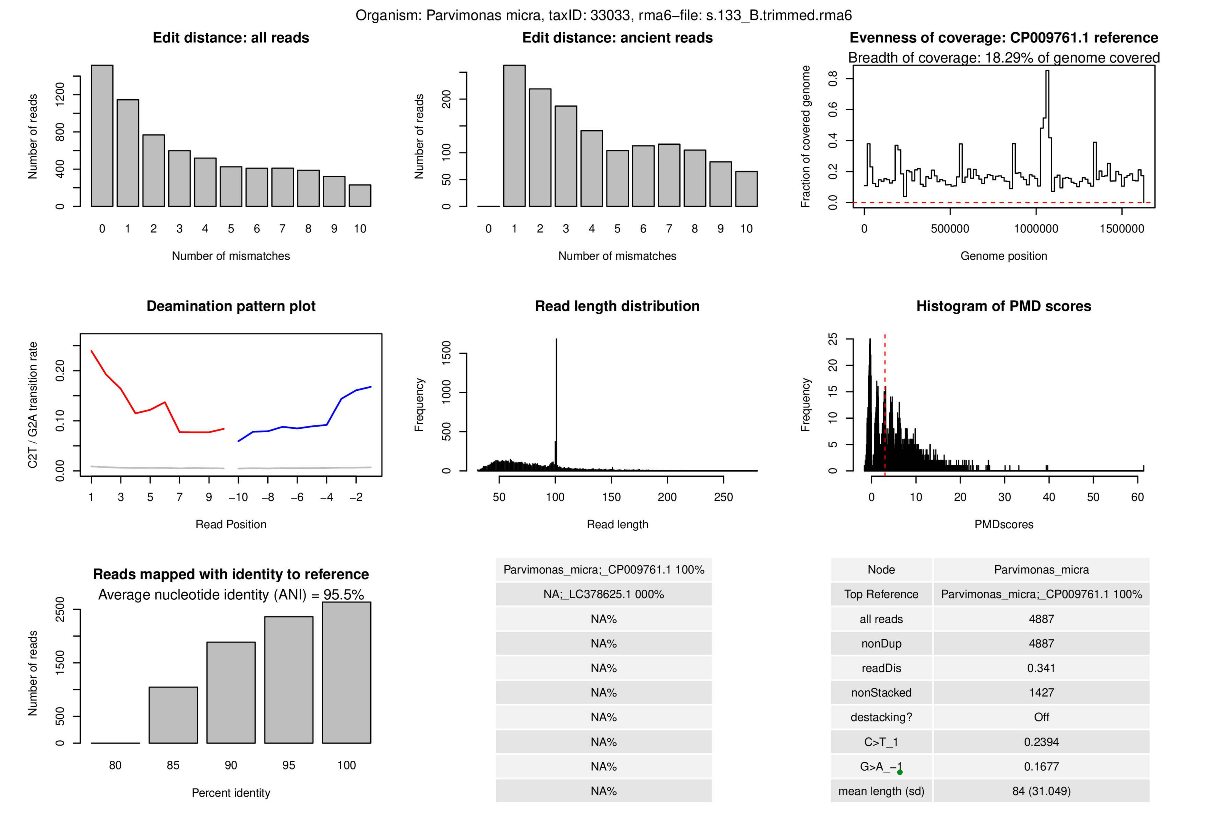


| |
|---|
| Parvimonas_micra;_CP009761.1 100% |
| NA;_LC378625.1 000% |
| NA% |
| NA% |
| NA% |
| NA% |
| NA% |
| NA% |
| NA% |
| NA% |

| | |
|---|---|
| Node | Parvimonas_micra |
| Top Reference | Parvimonas_micra;_CP009761.1 100% |
| all reads | 4887 |
| nonDup | 4887 |
| readDis | 0.341 |
| nonStacked | 1427 |
| destacking? | Off |
| C>T_1 | 0.2394 |
| G>A_-1 | 0.1677 |
| mean length (sd) | 84 (31.049) |

**Supplementary Fig. 15. Authentication plots for *Parvimonas micra* in individual s.133_B.** The panels display edit distance distributions (all vs ancient reads), genome coverage breadth and evenness, terminal deamination rates, read length distribution, PMD score histogram, and percent identity of mapped reads. A summary table includes top reference matches and mapping statistics. The overall authentication score is 8.

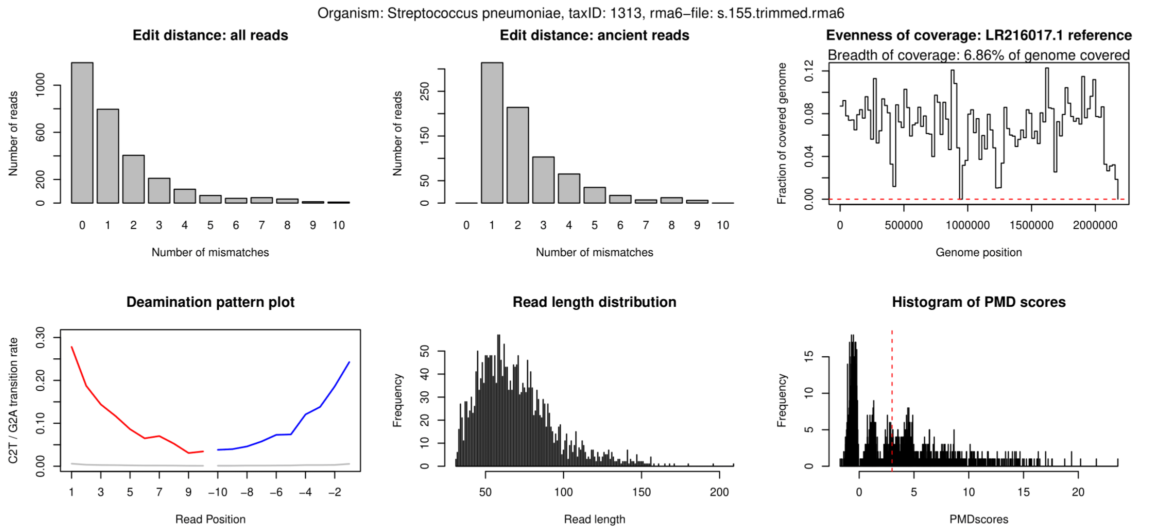


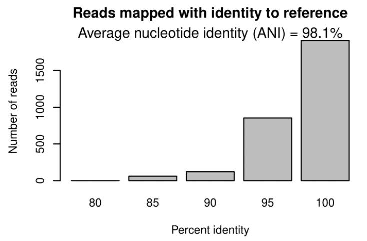


| |
|---|
| Streptococcus_pneumoniae;_LR216017.1 100% |
| NA;_Z83335.1 000% |
| NA;_X77249.1 000% |
| NA;_Z77726.1 000% |
| NA;_X67873.1 000% |
| NA;_X72967.1 000% |
| NA;_X67872.1 000% |
| NA;_Z77727.1 000% |
| NA;_Z79691.1 000% |
| NA;_X67868.1 000% |

| Node | Streptococcus_pneumoniae |
|---|---|
| Top Reference | Streptococcus_pneumoniae;_LR216017.1 100% |
| all reads | 1666 |
| nonDup | 1666 |
| readDis | 0.795 |
| nonStacked | 1243 |
| destacking? | Off |
| C>T_1 | 0.2782 |
| G>A_-1 | 0.2429 |
| mean length (sd) | 70 (24.658) |

**Supplementary Fig. 16. Authentication plots for *Streptococcus pneumoniae* in individual s.155.** The panels display edit distance distributions (all vs ancient reads), genome coverage breadth and evenness, terminal deamination rates, read length distribution, PMD score histogram, and percent identity of mapped reads. A summary table includes top reference matches and mapping statistics. The overall authentication score is 9.

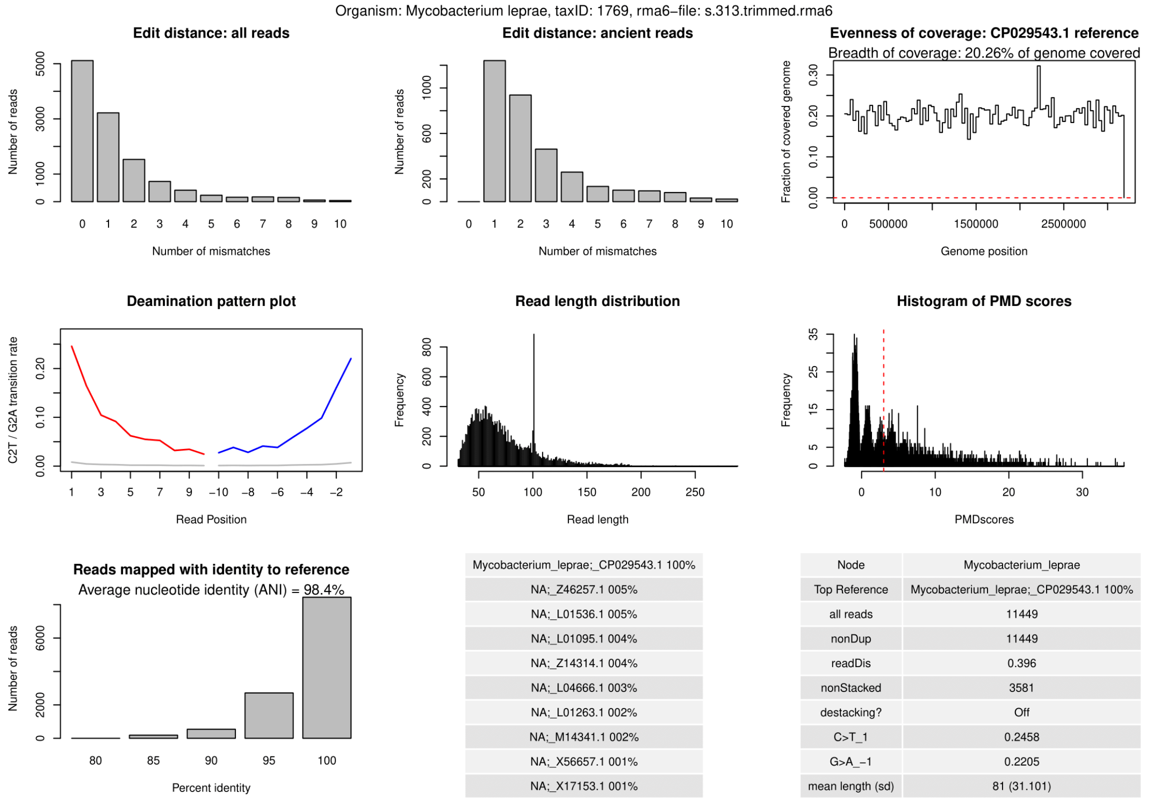


| |
|---|
| Mycobacterium_leprae;_CP029543.1 100% |
| NA;_Z46257.1 005% |
| NA;_L01536.1 005% |
| NA;_L01095.1 004% |
| NA;_Z14314.1 004% |
| NA;_L04666.1 003% |
| NA;_L01263.1 002% |
| NA;_M14341.1 002% |
| NA;_X56657.1 001% |
| NA;_X17153.1 001% |

| | |
|---|---|
| Node | Mycobacterium_leprae |
| Top Reference | Mycobacterium_leprae;_CP029543.1 100% |
| all reads | 11449 |
| nonDup | 11449 |
| readDis | 0.396 |
| nonStacked | 3581 |
| destacking? | Off |
| C>T_1 | 0.2458 |
| G>A_−1 | 0.2205 |
| mean length (sd) | 81 (31.101) |

**Supplementary Fig. 17. Authentication plots for *Mycobacterium leprae* in individual s.313.** The panels display edit distance distributions (all vs ancient reads), genome coverage breadth and evenness, terminal deamination rates, read length distribution, PMD score histogram, and percent identity of mapped reads. A summary table includes top reference matches and mapping statistics. The overall authentication score is 9.

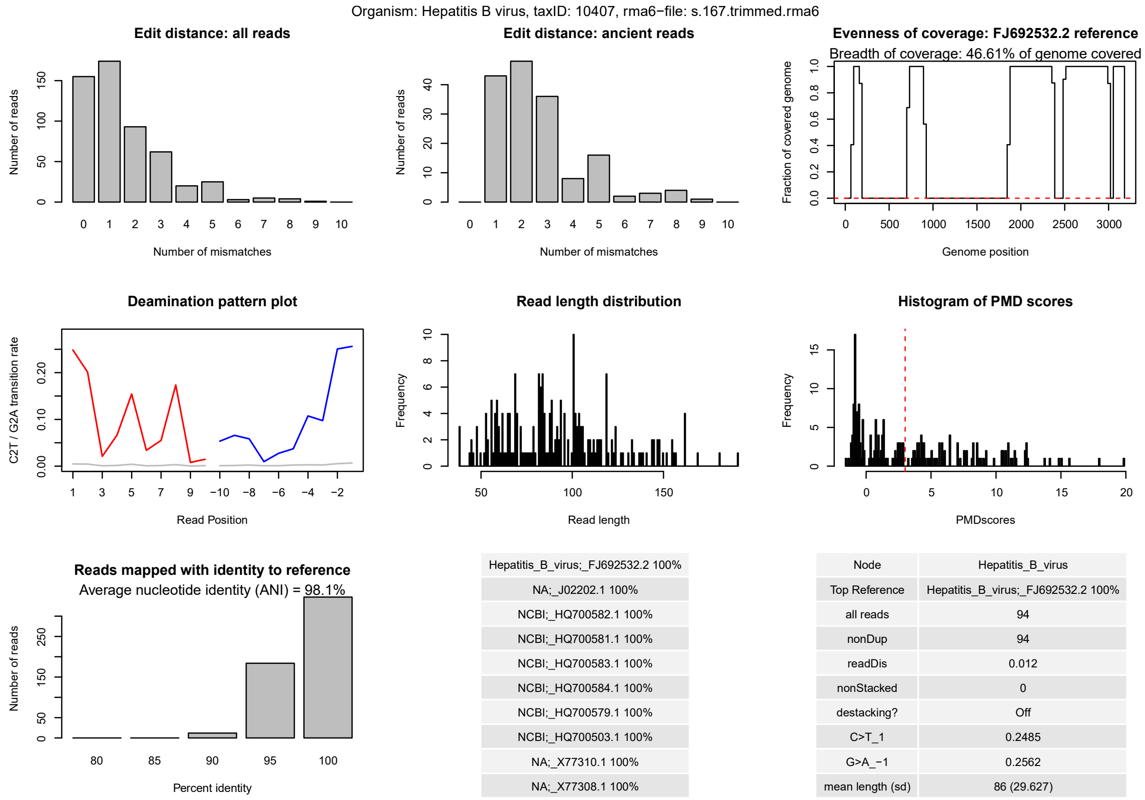


**Supplementary Fig. 18. Authentication plots for Hepatitis B virus in individual s.167.** The panels display edit distance distributions (all vs ancient reads), genome coverage breadth and evenness, terminal deamination rates, read length distribution, PMD score histogram, and percent identity of mapped reads. A summary table includes top reference matches and mapping statistics. The overall authentication score is 4.

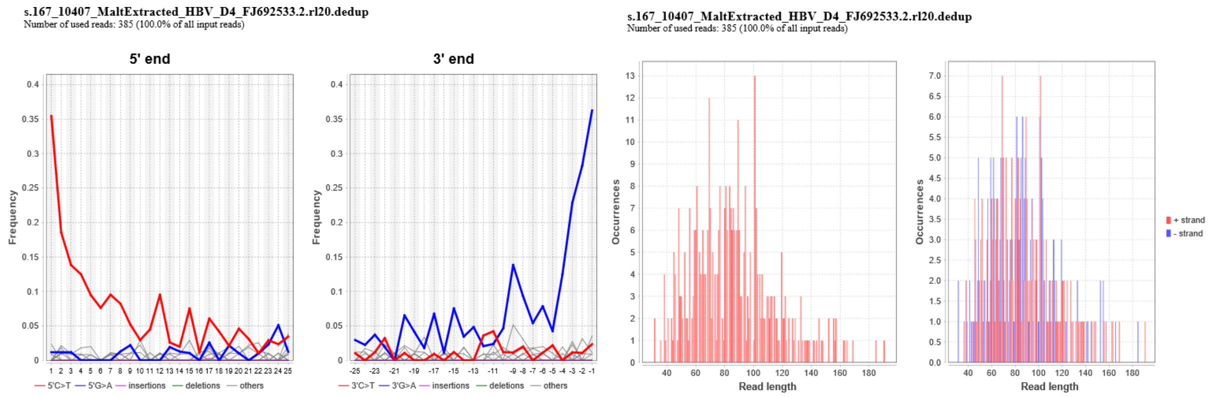


**Supplementary Fig. 19. Deamination plots and length plots of the reads assigned to HBV in s.167 and extracted with MaltExtract.** The closest reference genome according to BLAST was used for mapping (FJ692533.2).

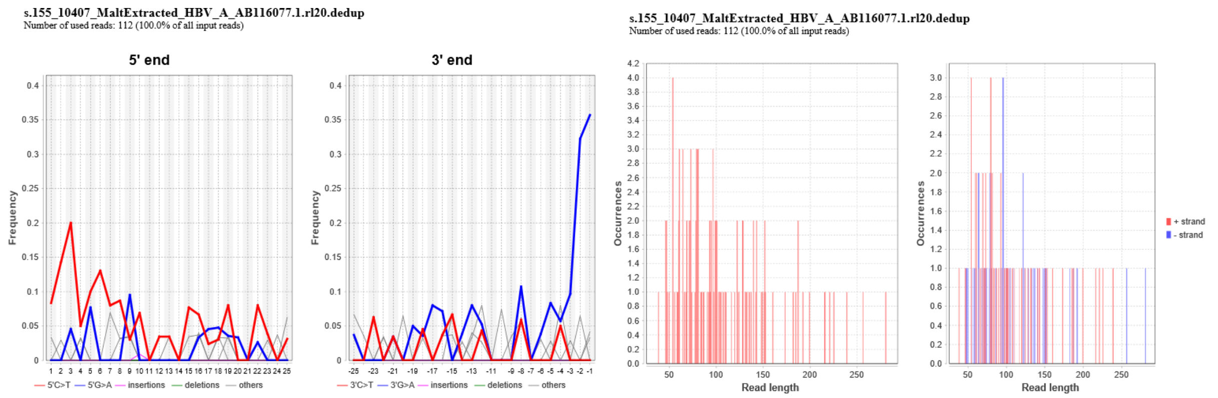


**Supplementary Fig. 20. Deamination plots and length plots of the reads assigned to HBV in s.155 and extracted with MaltExtract.** The closest reference genome according to BLAST was used for mapping (AB116077.1).

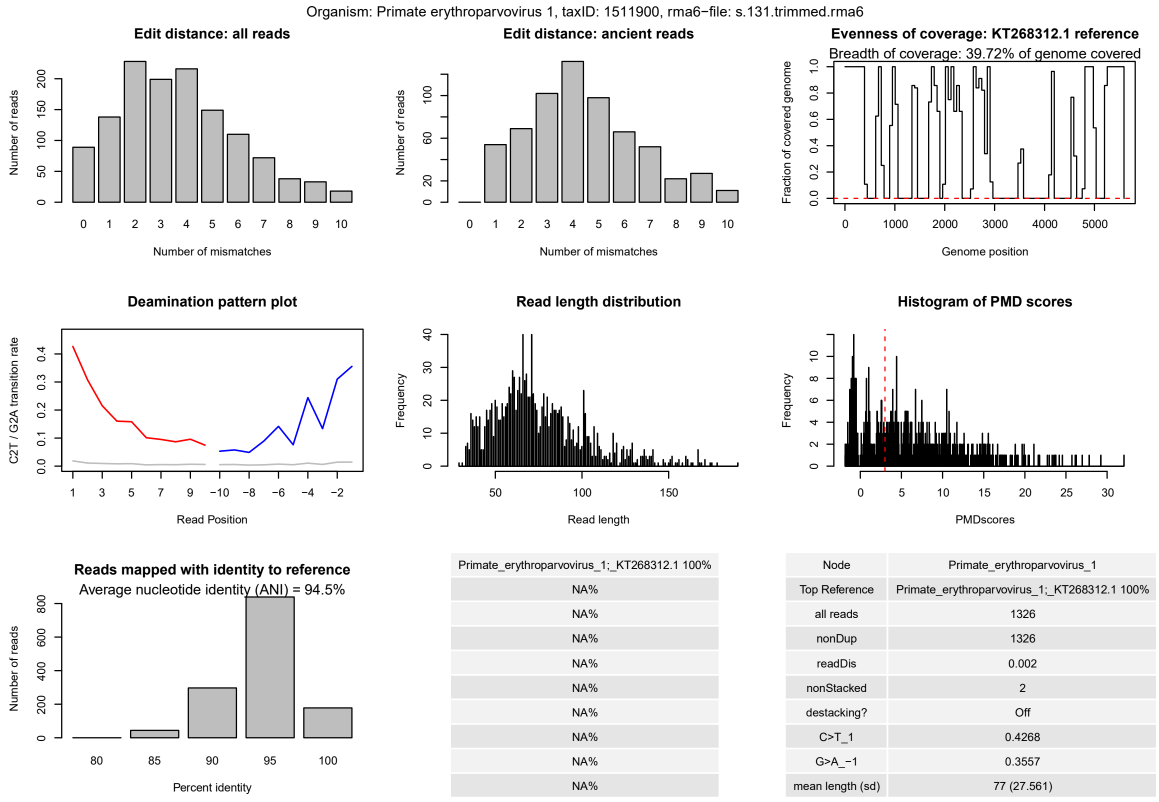


**Supplementary Fig. 21. Authentication plots for human parvovirus B19, alias primate erythroparvovirus 1, in individual s.131.** The panels display edit distance distributions (all vs ancient reads), genome coverage breadth and evenness, terminal deamination rates, read length distribution, PMD score histogram, and percent identity of mapped reads. A summary table includes top reference matches and mapping statistics. The overall authentication score is 4.

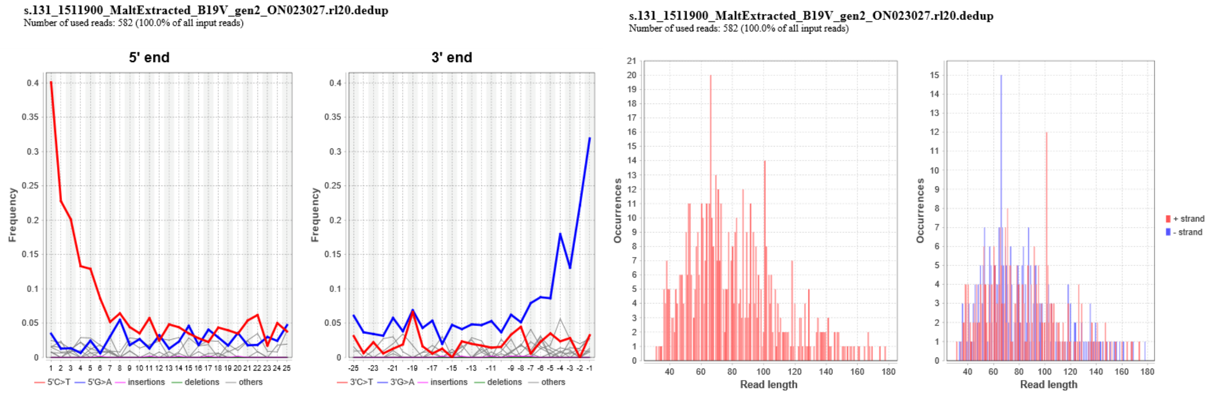


**Supplementary Fig. 22. Deamination plots and length plots of the reads assigned to B19V in s.131 and extracted with MaltExtract.** The closest complete reference genome according to BLAST was used for mapping (ON023027.1).

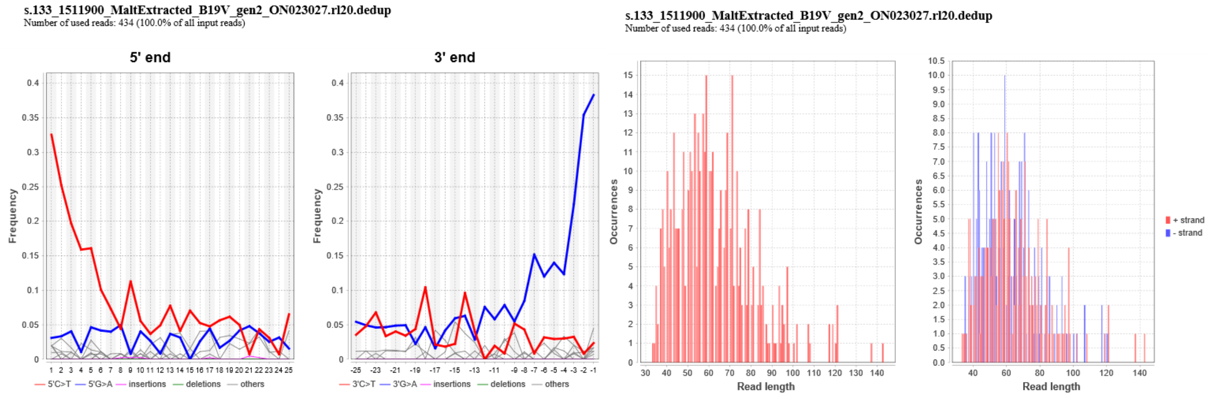


**Supplementary Fig. 23. Deamination plots and length plots of the reads assigned to B19V in s.133 and extracted with MaltExtract.** The reference genome ON023027.1 was used for mapping.

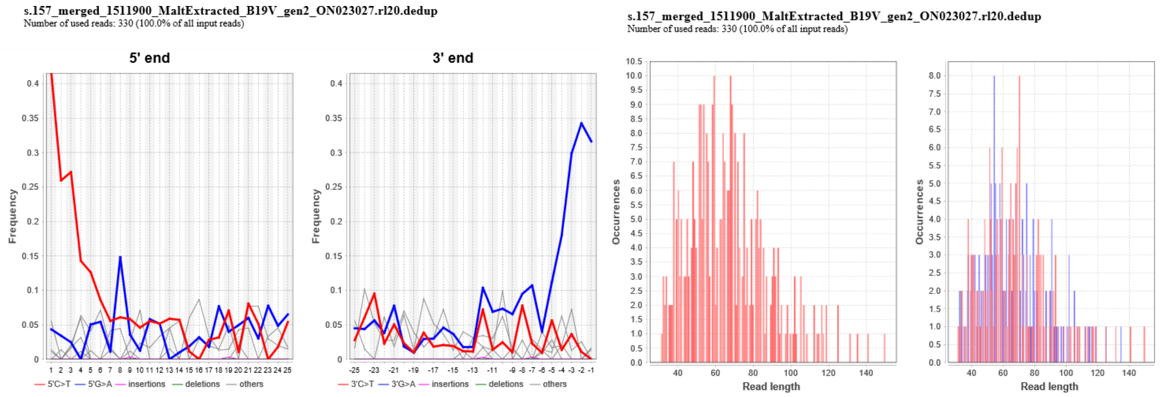


**Supplementary Fig. 24. Deamination plots and length plots of the reads assigned to B19V in s.157 and extracted with MaltExtract.** The reference genome ON023027.1 was used for mapping.

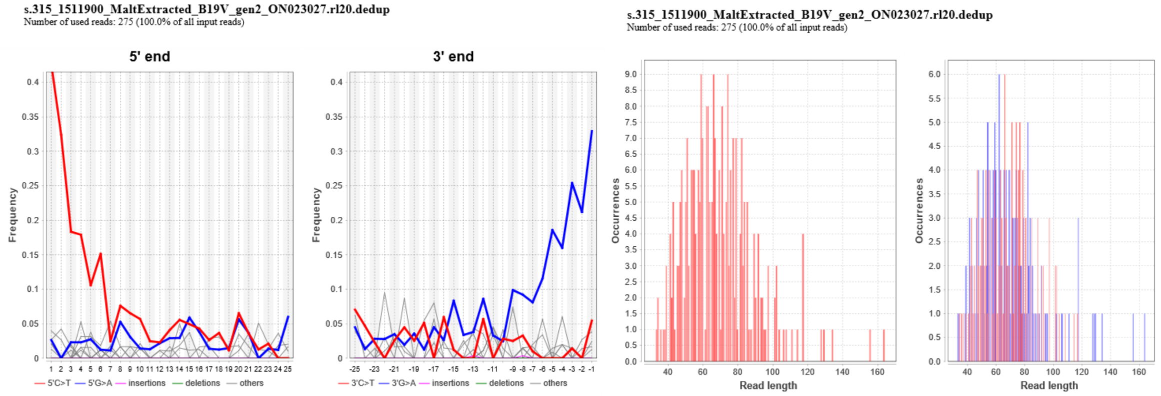


**Supplementary Fig. 25. Deamination plots and length plots of the reads assigned to B19V in s.315 and extracted with MaltExtract.** The reference genome ON023027.1 was used for mapping.

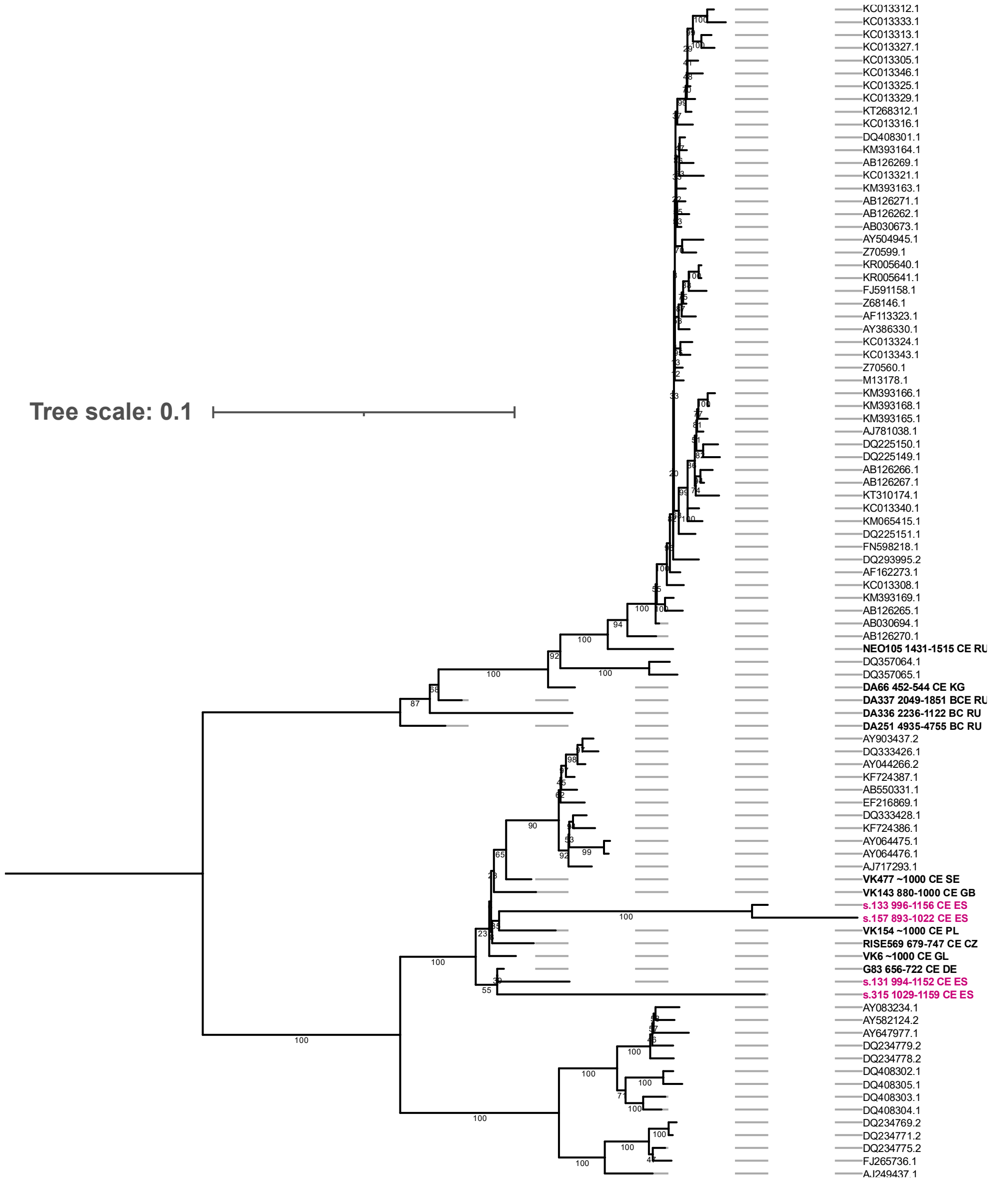


**Supplementar Fig. S26. Whole-genome maximum-likelihood (ML) phylogeny of human parvovirus B19.** The Ibiza samples (s.131, s.133, s.157 and s.315) are highlighted in red and fall within genotype 2. Ancient genomes are shown in bold with sample ID and country; modern samples are labelled with their GenBank accession numbers. Bootstrap values indicate branch support. The tree was reconstructed using the TIM3+F+R3 model and 100 bootstrap replicates and was midpoint-rooted.

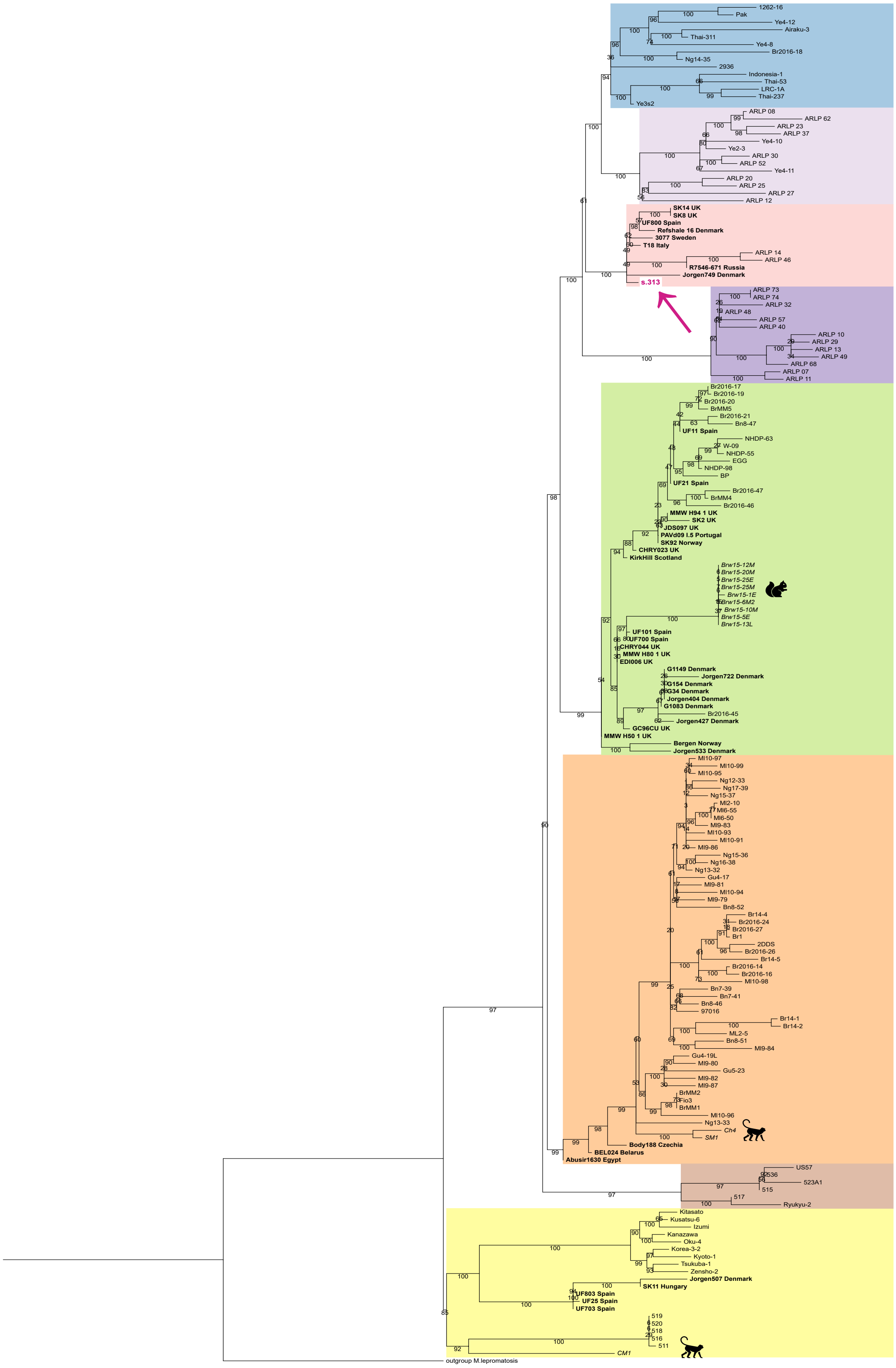


**Supplementary Fig. 27. Maximum-likelihood (ML) phylogeny of *Mycobacterium leprae*.** The Ibiza genome (s.313) is highlighted in red and falls within clade 2F. Ancient genomes are shown in bold with sample ID and country; those from animals are italicised; modern samples are labelled with the sample ID only. Bootstrap values indicate branch support. The tree was inferred from high-quality informative SNPs (≥3X coverage, genotype quality ≥30, base frequency ≥90%) retained at positions covered in ≥90% of the samples. *Mycobacterium lepromatosis* was used as the outgroup. The phylogeny was reconstructed using the GTR+F model and 100 bootstrap replicates.

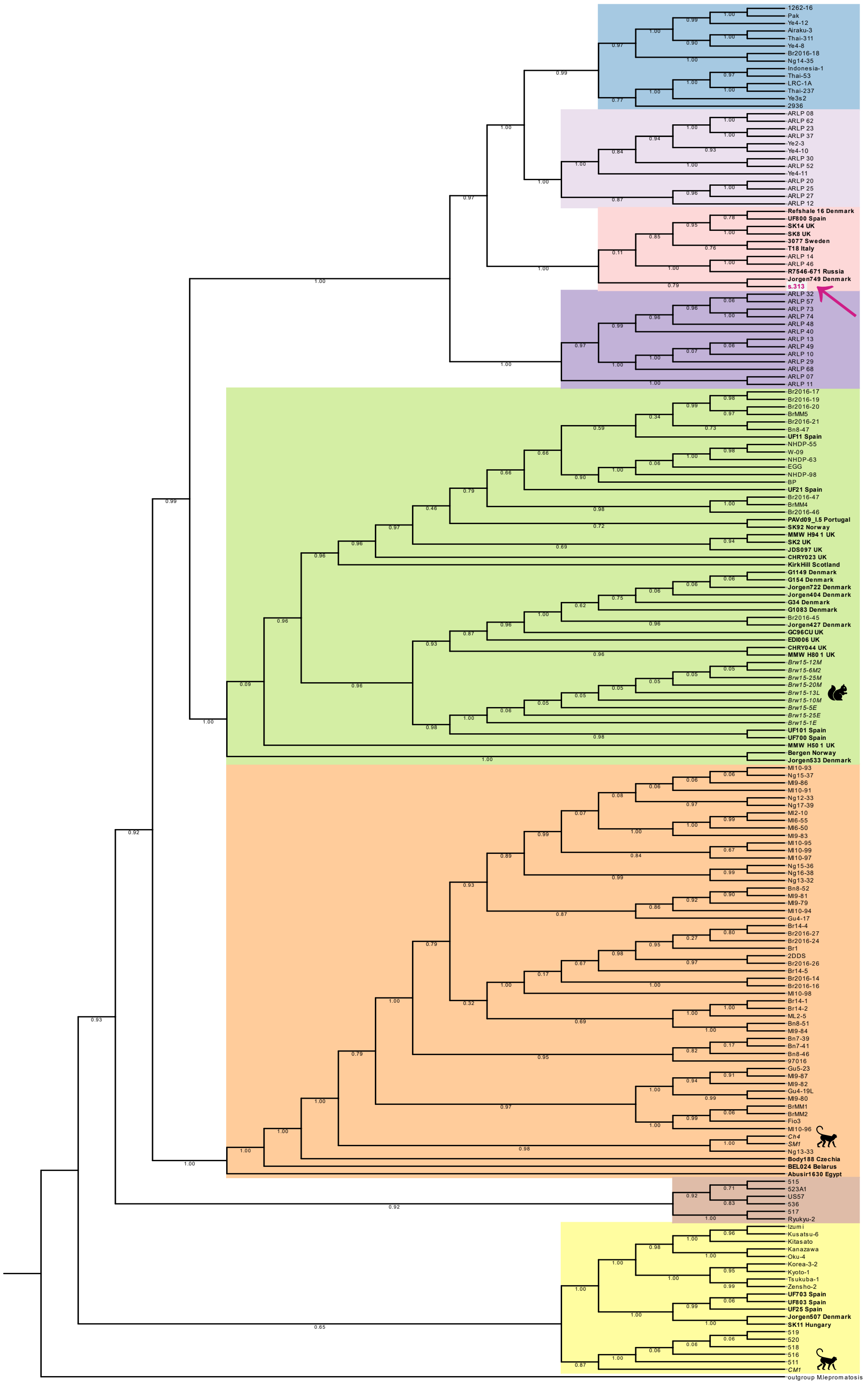


**Supplementary Fig. 28. Maximum Parsimony (MP) phylogeny of *Mycobacterium leprae.*** The Ibiza genome (s.313) is highlighted in red and falls within clade 2F. Ancient genomes are shown in bold with sample ID and country; those from animals are italicised; modern samples are labelled with the sample ID only. Bootstrap values indicate branch support. The tree was inferred from high-quality informative SNPs (≥3X coverage, genotype quality ≥30, base frequency ≥90%) retained at positions covered in ≥80% of the samples. *Mycobacterium lepromatosis* was used as the outgroup. The phylogeny was reconstructed with the Subtree-Pruning-Regrafting method with 500 bootstrap replicates.

## Supplementary References

1. Govan, E., Jackson, A. L., Inger, R., Bearhop, S. & Parnell, A. C. simmr: a package for fitting stable isotope mixing models in R. R package version (2023).
2. Alexander, M. M., Gerrard, C. M., Gutiérrez, A. & Millard, A. R. Diet, society and economy in late medieval Spain: stable isotope evidence from Muslims and Christians from Gandía, Valencia. *Am. J. Phys. Anthropol.* **156**, 263–273 (2015).
3. Pérez-Ramallo, P. *et al*. Unravelling social status in the first medieval military order of the Iberian Peninsula using isotope analysis. *Sci. Rep* **14**, 11074 (2024).
4. Dury, G. *et al*.The Islamic cemetery at 33 Bartomeu Vicent Ramon, Ibiza: investigating diet and mobility through light stable isotopes in bone collagen and tooth enamel. *Archaeol. Anthropol. Sci* **11**, 3913–3930 (2019).
5. Bronk Ramsey, C. *OxCal v4.4 Manual*. (Oxford Radiocarbon Accelerator Unit, University of Oxford, Oxford, 2021).
6. Reimer, P. J. *et al.* The IntCal20 Northern Hemisphere radiocarbon age calibration curve (0–55 cal kBP. *Radiocarbon* **62**, 725–757 (2020).
7. Siani, G. *et al.* Radiocarbon reservoir ages in the Mediterranean Sea and Black Sea. *Radiocarbon* **42**, 271–280 (2000).
8. Fellows Yates, B. J. A. *et al.* Community-curated and standardised metadata of published ancient metagenomic samples with AncientMetagenomeDir. *Sci. Data* **8, 31**, (2021).
9. Borry, M. *et al*. Facilitating accessible, rapid, and appropriate processing of ancient metagenomic data with AMDirT. *F1000Research* **12**, 926 (2024).
10. Schuenemann, V. J. *et al*. Genome-wide comparison of medieval and modern Mycobacterium leprae. *Science* **341**, 179–183 (2013).
11. Schuenemann, V. J. *et al*. Ancient genomes reveal a high diversity of Mycobacterium leprae in medieval Europe. *PLoS pathog.* **14**, 1006997 (2018).
12. Krause-Kyora, B. *et al*. Ancient DNA study reveals HLA susceptibility locus for leprosy in medieval Europeans. *Nat. Commun.* **9**, 1569 (2018).
13. Mendum, T. A. *et al*. Mycobacterium leprae genomes from a British medieval leprosy hospital: towards understanding an ancient epidemic. *BMC genomics* **15**, 1–8 (2014).
14. Fotakis, A. K. *et al*. Multi-omic detection of Mycobacterium leprae in archaeological human dental calculus. *Philos. Trans. R. Soc. B* **375**, 20190584 (2020).
15. Neukamm, J. *et al*. 2000-year-old pathogen genomes reconstructed from metagenomic analysis of Egyptian mummified individuals. *BMC bio.* **18**, 1–18 (2020).

16. Pfrengle, S. *et al*. Mycobacterium leprae diversity and population dynamics in medieval Europe from novel ancient genomes. *BMC biology* **19**, 1–18 (2021).
17. Urban, C. *et al*. Ancient Mycobacterium leprae genome reveals medieval English red squirrels as animal leprosy host. *Curr. Biol.* **34**, 2221–2230 (2024).
18. Avanzi, C. *et al*. Red squirrels in the British Isles are infected with leprosy bacilli. *Science* **354**, 744–747 (2016).
19. Avanzi, C. *et al*. Transmission of drug-resistant leprosy in Guinea-Conakry detected using molecular epidemiological approaches. *Clin. Infect. Dis.* **63**, 1482–1484 (2016).
20. Benjak, A. *et al*. Phylogenomics and antimicrobial resistance of the leprosy bacillus Mycobacterium leprae. *Nat.commun*.**9**, 352 (2018).
21. Blevins, K. E. *et al*. Evolutionary history of Mycobacterium leprae in the Pacific Islands. *Philos. Trans. R. Soc. B* **375**, 20190582 (2020).
22. Honap, T. P. *et al*. Mycobacterium leprae genomes from naturally infected nonhuman primates. *PLoS Negl. Trop. Dis.* **12**, 0006190 (2018).
23. Singh, P. *et al*. Insight into the evolution and origin of leprosy bacilli from the genome sequence of Mycobacterium lepromatosis. *Proc. Natl Acad. Sci. USA* **112**, 4459–4464 (2015).
24. Truman, R. W. *et al*. Probable zoonotic leprosy in the southern United States. N. Engl. J. Med. **364**, 1626–1633 (2011).
