# Supplementary material for: Analysis of medieval burials from Ibiza reveals genetic and pathogenic diversity during the Islamic period
Source: Nat Commun. 2026 Mar 26;17:2703. doi: 10.1038/s41467-026-70615-9 (PMC13021928; doi:10.1038/s41467-026-70615-9)
Supplement: Supplementary file 2 — Description of Additional Supplementary Files [file 41467_2026_70615_MOESM2_ESM.pdf]

Description of Additional Supplementary Files

File Name: Supplementary Data 1
Description: Summarizes the osteological, chronological, and genetic characteristics of all individuals analysed. It includes archaeological identifiers, estimated age-at-death and stature, radiocarbon dates (IOSACal and OxCal), skeletal element sampled, Y-chromosome haplogroups, sequencing metrics, biological sex, and damage patterns. It also reports mitochondrial and X-chromosome contamination estimates using multiple methods and confidence intervals

File Name: Supplementary Data 2
Description: Radiocarbon and stable isotope data for each individual, including tissue type, laboratory identifiers, $\delta^{13}C$ and $\delta^{15}N$ values, C:N ratios, and uncalibrated radiocarbon ages. It also details the calibration parameters used—calibration curves, software, and marine reservoir corrections—along with modelled dietary fractions (terrestrial vs. marine) and the resulting calibrated date ranges before and after applying marine reservoir effects

File Name: Supplementary Data 3
Description: Estimated kinship relationships among individuals analysed in this study

File Name: Supplementary Data 4
Description: Summarizes runs of homozygosity (ROH) per individual, including the number of SNPs analysed, the length of the longest ROH, and counts and cumulative lengths of ROH across different size thresholds (4, 8, 12, and 20 Mb), providing insights into individual inbreeding and population history

File Name: Supplementary Data 5
Description: Summarizes metagenomic classification of sequenced reads, including the species identified, sample source, proportion of reads assigned, coverage metrics, k-mer statistics, duplication levels, and validation of taxonomic assignments via BLAST, highlighting ambiguous or alternative top hits

File Name: Supplementary Data 6
Description: Summary of authentication metrics for *Streptococcus pneumoniae* based on KrakenUniq, aMeta authentication score, MaltExtract read count and BLASTn summary

File Name: Supplementary Data 7
Description: Summary of authentication metrics for *Mycobacterium leprae* based on KrakenUniq, aMeta authentication score, MaltExtract read count and BLASTn summary

File Name: Supplementary Data 8
Description: Summary of authentication metrics for Hepatitis B virus based on KrakenUniq, aMeta authentication score, MaltExtract read count and BLASTn summary.

File Name: Supplementary Data 9
Description: Summary of authentication metrics for Primate erythroparvovirus 1 based on KrakenUniq, aMeta authentication score, MaltExtract read count and BLASTn summary.
